# Comprehensive Characterization of Platelet-Enriched MicroRNAs as Biomarkers of Platelet Activation

**DOI:** 10.3390/cells11081254

**Published:** 2022-04-07

**Authors:** Teresa L. Krammer, Stephan Zeibig, Waltraud C. Schrottmaier, Anita Pirabe, Silvia Goebel, Andreas B. Diendorfer, Hans-Peter Holthoff, Alice Assinger, Matthias Hackl

**Affiliations:** 1 TAmiRNA GmbH, 1110 Vienna, Austria; teresa.krammer@tamirna.com (T.L.K.); andreas.diendorfer@tamirna.com (A.B.D.); 2 Department of Vascular Biology and Thrombosis Research, Center of Physiology and Pharmacology, Medical University of Vienna, Schwarzspanierstraße 17, 1090 Vienna, Austria; waltraud.schrottmaier@meduniwien.ac.at (W.C.S.); anita.pirabe@meduniwien.ac.at (A.P.); alice.assinger@meduniwien.ac.at (A.A.); 3 AdvanceCOR GmbH, Fraunhoferstr. 9a, D-82152 Martinsried, Germany; zeibig@advancecor.com (S.Z.); goebel@advancecor.com (S.G.); peter.holthoff@isarbioscience.de (H.-P.H.)

**Keywords:** platelet-enriched miRNAs, microRNA, biomarker, antiplatelet therapy, platelet activation, microvesicles, plasma miRNAs, thrombomiRs

## Abstract

Dysregulation of platelet function is causally connected to thrombus formation and cardiovascular diseases. Therefore, assessing platelet reactivity is crucial. However, current platelet function tests come with pitfalls, limiting clinical use. Plasma miRNA signatures have been suggested as novel biomarkers for predicting/diagnosing cardiovascular diseases and monitoring antiplatelet therapy. Here, we provide results from a comprehensive study on the feasibility of using circulatory platelet miRNAs as surrogate markers of platelet activation. We performed small RNA-Seq on different blood cell types to confirm known and identify novel platelet-enriched miRNAs and validated a panel of 16 miRNAs using RT-qPCR. To identify the main carrier of these blood-based platelet miRNAs, we enriched and analyzed distinct microvesicle populations. Platelets were stimulated with GPVI and P2Y12 agonists in vitro to monitor the release of the selected miRNAs following activation. Finally, the miRNA panel was also measured in plasma from mice undergoing the Folts intervention (recurrent thrombus formation in the carotid artery). Applying an unbiased bioinformatics-supported workflow to our NGS data, we were able to confirm a panel of previously established miRNA biomarker candidates and identify three new candidates (i.e., miR-199a-3p, miR-151a-5p, and miR-148b-3p). Basal levels of platelet-derived miRNAs in plasma were mainly complexed with proteins, not extracellular vesicles. We show that changes in miRNA levels due to platelet activation are detectable using RT-qPCR. In addition, we highlight limitations of studying the in vitro release of miRNAs from platelets. In vivo thrombosis resulted in significant elevations of platelet-derived miRNA levels in mice. In conclusion, we provide in-depth evidence that activated platelets release miRNAs, resulting in measurable changes in circulatory miRNA levels, rendering them promising biomarker candidates.

## 1. Introduction

Platelets are the second most abundant blood cell type and play a pivotal role in hemostasis. Dysregulation of platelet function is causally connected to thrombosis and contributes to ischemia in patients with cardiovascular diseases (CVDs). To predict cardiovascular events and guide antiplatelet treatment decisions, monitoring platelet function is crucial. However, current platelet function tests (PFTs) have limitations, impeding use in clinical routine. Biomarkers to assess the in vivo state of platelets would therefore be highly beneficial. Cell-free microRNAs (miRNAs) in the blood are emerging biomarker candidates of CVDs [1]. MiRNAs are single-stranded, approximately 22 nt long non-coding RNAs that fine-tune protein expression. MiRNAs can be stably detected in plasma [2] and show cell- and tissue-specific enrichment [3,4], rendering them attractive biomarker candidates. Despite lacking a nucleus, platelets are capable of processing precursor-miRNAs (pre-miRNAs) [5] and contain up to 750 different miRNA species [6]. The platelet miRNome is dynamic. Even though the majority of the miRNA content in platelets is inherited from megakaryocytes [7,8], platelets are also capable of RNA uptake from the environment [9]. One decade ago, the first study linking miRNA levels in platelets to platelet reactivity was published [10], paving the way for numerous investigations into platelet miRNAs as biomarkers of platelet activation, circumventing the pitfalls of standard PFTs such as insufficient standardization or the need for fresh blood. Several platelet-derived miRNAs (thrombomiRs) in blood have been shown to be responsive to antiplatelet treatment (in healthy volunteers as well as in patients suffering from symptomatic carotid atherosclerosis) [11]. In this small but groundbreaking study, plasma miR-223, miR-191, miR-150, and miR-126 were significantly decreased in response to platelet inhibition [11]. These findings were further corroborated by a study reporting that levels of platelet-enriched miRNAs in the circulation are correlated with the platelet activation status in the general population (Bruneck cohort) as well as patients suffering from acute coronary syndrome (ACS) [12]. Recently, a study reported that in vitro agonist stimulation of extracted platelets in buffer results in the release of miRNAs, independently of the agonist used. The authors report 46 different secreted miRNAs with miR-223-3p being the most abundant [13]. However, the authors did not include an unstimulated control, raising the question of whether miRNAs could have been released through sample handling rather than agonist stimulation [13].

Despite substantial evidence demonstrating the high potential of platelet miRNAs as biomarkers of platelet activation, there is currently no comprehensive study on the feasibility and additional informational content gained through miRNA analysis. Therefore, our objective was to perform an in-depth, unbiased analysis of whether plasma levels of platelet-derived miRNAs can be used as surrogate markers of platelet activation. For this we:

1. Sequenced various blood cell types, biofluids, and platelet supernatant to identify novel and confirm known platelet-enriched miRNAs;
2. Characterized the in vitro release of miRNAs upon platelet activation including the EV and protein fractions to which miRNAs can be associated;
3. Used a mouse model of in vivo thrombogenesis (Folts intervention) to translate our findings from in vitro to in vivo.

## 2. Materials and Methods

### 2.1. Blood Collection from Healthy Volunteers and Preparation of Blood Cells and Plasma

Blood was drawn from healthy volunteers devoid of any medication for at least 10 days using a 21 G needle from the antecubital vein and anticoagulated with CTAD (BD vacutainer). All volunteers gave informed consent, and the study was approved by the local ethics committee (1548/2020). To obtain the different blood cells, blood was centrifuged for 20 min at 125× *g* at room temperature (RT) to obtain platelet-rich plasma (PRP) in the supernatant, buffy coat from the intermediate fraction (also subsequently referred to as “leukocytes”), and erythrocytes from the bottom of the tube. Part of each fraction was immediately frozen at −80 °C until further use. Fresh PRP was further processed to obtain either:

(a) Gel-filtrated platelets using a Sepharose 4B column with HEPES-Tyrode buffer containing 0.5% human serum albumin [14];
(b) Washed platelets (centrifuged for 2 min, 2000× *g*, RT) in the presence of 0.8 µM prostacyclin (PGI_2_) (Sigma–Aldrich, St. Louis, MO, USA) and washed once in phosphate-buffered saline (PBS) (w/o: Ca^2+^ and Mg^2+^) containing PGI_2_ (0.8 µM).

The supernatant was carefully discarded, and the platelet pellet was frozen at −80 °C until further processing. To gain the supernatant of the stimulated platelets, washed platelets were activated with 25 ng/mL convulxin. The supernatant was frozen at −80 °C until further processing.

### 2.2. Microvesicle Enrichment and Depletion Using MACS Beads 

The CTAD-anticoagulated blood was centrifuged for 10 min at 2000× *g* to generate platelet-poor plasma (PPP), and the supernatant was re-centrifuged for 20 min at 2000× *g* to clear cellular debris and obtain platelet-free plasma (PFP). To isolate microvesicles (MVs), PFP was centrifuged at 80,000× *g* for 1 h, and the MV-containing pellet was resuspended in filtered PBS. The MVs were then further separated using MojoSort Streptavidin Nanobeads (BioLegend Europe B.V., Uithoorn, Netherlands) in combination with biotin Annexin V (BioLegend) for enrichment and depletion of phosphatidylserine (PS)-positive MVs, biotin anti-CD45 (BioLegend) to enrich and deplete for leukocyte-derived MVs, biotin anti-CD235b (BioLegend) to deplete and enrich erythrocyte-derived MVs, and biotin anti-CD31 to enrich and deplete platelet- and endothelial-derived MVs. Platelet-derived MVs were specifically enriched/depleted by using MACS cell separation anti-CD61 microbeads (Miltenyi Biotec, Bergisch Gladbach, Germany).

### 2.3. Vesicle Isolation using Size-Exclusion Chromatography

PPP was centrifuged at 12,000× *g* for 5 min at 4 °C, and 150 μL of the supernatant were loaded onto qEV single columns (70 nm, Izon, Lyon, France). The columns were used according to the manufacturer’s instructions. Six hundred microliters EV fraction and 4 mL protein fraction were collected and subsequently concentrated using Amicon tubes (10 kDa, Merck, Darmstadt, Germany) according to the recommendations of the manufacturer. As soon as volumes were below 200 μL, the sample was collected and diluted to exactly 200 μL with nuclease-free water (NFW).

### 2.4. In Vitro Activation of Platelets

Blood was collected from healthy donors devoid of medication for at least 10 days using a 21 G needle (Sarstedt, Nümbrecht, Germany) from the antecubital vein and anticoagulated using citrate (Sarstedt). All volunteers gave informed consent. PRP was generated by centrifugation of whole blood (150× *g*, 20 min, no brakes), and 0.1 μg/mL PGI_2_ was added to each sample to avoid artificial platelet activation.

For the activation of platelets in buffer, PRP was centrifuged (500× *g*, 12 min). Platelets were resuspended twice in equal volumes of 0.9% NaCl and 0.1 μg/mL PGI_2_. After each washing step, samples were centrifuged at 500× *g*, and the supernatant was discarded. Following the last centrifugation step, platelets were resuspended in one-third of the initial volume in 0.9% NaCl. To avoid artificial platelet activation, 0.1 μg/mL PGI_2_ were added to each sample. Thereafter, platelets were counted and diluted to 500,000 platelets/μL. Five hundred microliters of washed platelets were activated for 30 min at 37 °C without stirring using 1A5 (GPVI receptor agonist), ADP, or 1 μg/mL PGI_2_ (negative control). After incubation, 1 μg/mL PGI_2_ was added, and all samples were centrifuged at 1000× *g* for 10 min. The supernatant was centrifuged again for 10 min at 10,000× *g*. Two hundred microliters of platelet-free supernatant were stored at −80 °C until further processing. 

For activation of platelets in PRP, 500 μL aliquots were used for the addition of agonists (1A5, ADP, or equivalent volume of 0.9% NaCl). Samples were left at RT for 30 min. Following the activation step, 1 μg/mL PGI_2_ was added and samples were centrifuged as described above to generate PPP.

### 2.5. Measurement of Platelet Activation Markers

For the measurement of P-selectin (CD62P) expression, platelets (from 5 μL PRP or washed platelets) were labeled after incubation with the agonists using APC-labeled mouse anti-human-CD61 antibody (Millipore), PE-labeled mouse anti-human-CD62P antibody (BD Pharmingen), and the appropriate isotype control (mouse IgG1 PE, Santa Cruz Biotechnology). Platelet suspensions were added to the antibody dilutions (1:20) and incubated for 20 min at RT, protected from light. Samples were then measured using a BD FACSCanto^TM^ II Flow Cytometry System (BD Biosciences) (Figure S7).

For platelet aggregation, 0.1 µg/mL PGI_2_ were added immediately after blood collection, and the blood was incubated for 10 min at RT. Then, the blood was centrifuged for 15 min at 150× *g* without a brake, and the supernatant was carefully removed without disturbing the milky interface layer, generating PRP. For background calibration, one aliquot of PRP was centrifuged at 10,000× *g* to pellet residual platelets and gain PPP. Aggregation measurements were performed on an APACT 4004 aggregometer, pre-heated to 37 °C. Stirring was set to 1000 rpm. One hundred and fifty-six microliters of PRP were used for each measurement, and 4 µL of supplement/PBS control were added before starting the data collection (160 µL total volume). Light transmission at 740 nm was determined, and data were collected for 420 s (Figure S7).

### 2.6. RNA Extraction and qPCR Analysis

Total RNA was extracted from plasma (i.e., PPP and PRP) or frozen blood cells using the miRNeasy Mini Kit (Qiagen, Venlo, The Netherlands). Input volumes varied between 20 and 200 µL (Table 1); all samples were diluted to 200 µL with NFW. Samples were thawed at RT and biofluids were centrifuged at 12,000× *g* for 5 min at 4 °C to remove cellular debris. Samples were homogenized with 1000 µL Qiazol and rigorous mixing for 10 s (1 min for cells), followed by 10 min incubation at RT. Two hundred microliters of chloroform were added, and lysates were again mixed and left at RT for 3 min. Subsequently, samples were centrifuged at 12,000× *g* for 15 min at 4 °C. For plasma samples, precisely 650 µL aqueous phase were transferred to fresh tubes, and 7 µL glycogen (5 mg/mL) were added to improve precipitation. For cells, the entire aqueous phase was used and diluted to 650 µL with NFW. A QIAcube liquid handling robot was utilized for binding to RNeasy Mini Spin Columns and washing steps with RPE and RWT buffers. Total RNA was eluted in 30 µL NFW and stored at −80 °C until further analysis.

Reverse transcription was carried out using the miRCURY RT kit (Qiagen), following the manufacturer’s instructions. Two microliters of total RNA were input into 10 μL reactions. For blood cells, we calculated back to 10.2 ng total RNA input for all samples. Quantitative PCR (qPCR) was performed using the miRCURY SYBR^®^ Green Master Mix and commercial LNA-enhanced miRNA assays (Qiagen) (only mature miRNAs are detected). The final cDNA dilution was 1:100 (10 μL PCR reaction). To ensure the quality of the generated data, synthetic spike-ins (all Qiagen) were added in equimolar amounts before the respective step in the workflow to assess the efficacy of RNA isolation (UniSp4), reverse transcription (cel-miR-39-3p), and PCR (UniSp3). qPCRs were performed on a LightCycler 480 II (Roche, Basel, Switzerland) or a LightCycler 96 (Roche) with the following settings: 95 °C for 2 min (activation), 45 cycles of 95 °C for 10 s, and 56 °C for 60 s. Melting curves were generated using continuous acquisition between 55 and 98 °C. Cq values were calculated using the 2nd derivative maximum method (LC480, Roche v1.5.1.62) or a combination of the 2nd derivative maximum and the fits point method (LC96, Roche v1.1).

Hemolysis of the samples was assessed using the ratio of miR-23a-3p and miR-451a as previously described [15]. Hemolytic samples were excluded from further analysis. To ensure the quality of the obtained data, RNA, cDNA, and PCR spike-ins were assessed for all samples (summarized for all RT-qPCR experiments in Figure S8). Normalization of Cq values was performed using the RNA spike-in control as internal standard [16]. ΔCq values were calculated by subtracting the RNA spike-in Cq from the miRNA Cq:

ΔCq = Cq_(RNA spike-in)_ − Cq_(target miRNA)_ (1)

### 2.7. Absolute Quantification of miRNAs

RT-qPCR was carried out as described above. Commercial LNA-enhanced primer assays for hsa-miR-223-3p, hsa-miR-199a-3p, hsa-miR-191-5p, hsa-miR-151a-5p, hsa-miR-148b-3p, hsa-miR-126-3p, and hsa-miR-21-5p (all Qiagen) were used. For each miRNA, 5′phosphorylated RNA oligos were synthesized (Table 2). The RNA mimics (IDT) were diluted corresponding to 10^9^ to 10^3^ copies/qPCR well and included in the RT reaction. The obtained Cq values within the linear range were used to fit a line to the data from each dilution series. Using the standards curves, raw Cq values from the samples were converted to copies/platelet or copies/μL plasma.

### 2.8. Small RNA Library Preparation 

Ten nanograms of total RNA (blood cells) and 2 μL total RNA (biofluids) were used for small RNA library preparations. The CleanTag Small RNA Library Preparation Kit (TriLink Biotechnologies, San Diego, CA, USA) was used according to the manufacturer’s specifications. Adapter-ligated libraries were amplified (18 cycles for blood cells, 24 cycles for biofluids) utilizing barcoded Illumina reverse primers together with the Illumina forward primer. The QIAQuick PCR Purification Kit (Qiagen) was then used to purify the PCR product. Two pools, each containing 20 libraries, were prepared at equimolar rates on the basis of a DNA1000 bioanalyzer run (Agilent vB.02.10.SI764). The pools were then processed with the Blue Pippin system (Sage Science v6.40) using 3% agarose size selection cassettes, following the manufacturer’s instructions (size range: 125–160 bp; target sequence: 142 bp; range flag: broad; internal standards). Sequencing was performed on an Illumina HiSeqV4 with 50 bp single-end reads.

### 2.9. Folts Intervention

For the Folts intervention experiments, male and female C57BL/6J mice (8–14 weeks) were anesthetized with medetomidine (0.5 mg/kg), midazolam (5 mg/kg), fentanyl (0.05 mg/kg), and isoflurane. The ventral neck area was shaved and disinfected. Animals were fixed in a supine position on a warming pad with constant monitoring of body temperature. The ventral neck was cut from sternum to chin angle. A surgical suture (6/0) was placed around the left arteria carotis communis (CCA), and both ends were threaded through a 2–3 mm long silicone tube. Both suture ends were fixed with a hemostatic clamp. Distal to the tied suture, a flow probe was applied to measure cyclic flow reductions (CFRs). The CCA was constricted by pushing down the silicone tube with the hemostat over filament until the flow decreased by 10–20%. The intima was injured by moving the suture in the direction of the sternum and clamping the vessel using a microneedle holder; the suture was positioned on top of the injury, inducing the formation and subsequent dislodging of platelet-rich thrombi, causing CFRs. If no flow reductions occurred within 5 min, clamping with the microneedle holder was repeated. Measurement of blood flow commenced as soon as CFRs occurred every 45–80 s (1.33–0.75 CFRs/min). Measurements were stopped after 30 min. Blood was drawn from the tail vein before the intervention and intracardially after the intervention.

### 2.10. Statistical Analysis

#### 2.10.1. NGS Data

The overall quality of the next-generation sequencing (NGS) data was evaluated automatically and manually with FastQC v0.11.8 [17] and MultiQC v1.7 [18]. Reads from all passing samples were adapter trimmed and quality filtered using Cutadapt v2.3 [19] and filtered for a minimum length of 17 nt. Mapping steps were performed with Bowtie v1.2.2 [20] and miRDeep2 v2.0.1.2 [21], whereas reads were mapped first against the genomic reference GRCh38.p12 provided by Ensembl [22], allowing for two mismatches and, subsequently, miRBase v22.1 [23], filtered for miRNAs of hsa only, allowing for one mismatch. For a general RNA composition overview, non-miRNA mapped reads were mapped against RNAcentral [24] and then assigned to various RNA species of interest. Statistical analysis of preprocessed NGS data was done with R v3.6 and the packages pheatmap v1.0.12, pcaMethods v1.78, and genefilter v1.68. Differential expression analysis with edgeR v3.28 [25] used the quasi-likelihood negative binomial generalized log-linear model functions provided by the package. The independent filtering method of DESeq2 [26] was adapted for use with edgeR to remove low abundant miRNAs and, thus, optimize the false discovery rate (FDR) correction.

Data are presented as boxplots ranging from minimum to maximum and displaying the median. For qPCR validation, blood cells were cDNA spike-in normalized (ΔCq_cel-miR-39_) and plasma was RNA spike-in normalized (ΔCq_UniSp4_).

#### 2.10.2. Microvesicle Enrichment and Depletion

Normalized data (ΔCq_UniSp4_) were linearized and relative expression (%) compared to the control (PPP) was calculated.

#### 2.10.3. In Vitro Activation of Platelets

Data are presented as mean ± SD. Depending on whether values were missing in the respective data set, repeated-measures one-way ANOVA or a mixed-effects model with Sidak or Tukey multiple comparisons tests were calculated.

All statistical analyses (excluding NGS data) were performed using GraphPad Prism v9.2.0 (www.graphpad.com, accessed on 20 February 2022). *p*-Values < 0.05 were considered statistically significant.

## 3. Results

### 3.1. NGS of Blood Cells, Plasma, and the Supernatant of Activated Platelets Confirms a Previously Established Panel of Platelet-Enriched miRNAs as Promising Biomarker Candidates of Platelet Activation

**Figure 1:**
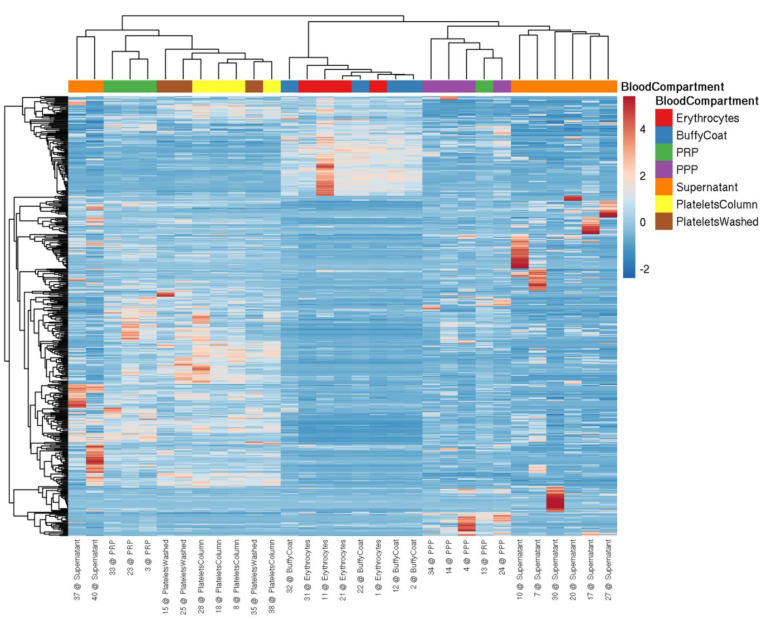
Heatmap visualizing miRNA levels determined by NGS in blood cells and plasma from four healthy volunteers. miRNA profiles are suitable to distinguish between different blood cells (i.e., erythrocytes, leukocytes (buffy coat), and platelets (isolation via columns or washing steps)), plasma (i.e., PRP and PPP), and the supernatant of convulxin-stimulated platelets. The data were scaled using the unit variance method, and clustering was performed by applying the average method of the pheatmap package, calculating the distances as correlations.

First, we set out to verify the enrichment of a previously established panel of miRNAs (Table 3) in platelets compared to other blood cell types using small RNA NGS analysis. In addition, PPP and PRP samples were included as well as supernatant of in vitro activated platelets. In blood cells between ~25% of total quality-filtered reads (platelets) and 90% (red blood cells) mapped to miRNAs (Figure S1A), we identified >350 distinct mature miRNAs with a read count >10 in platelets (Figure S1B). The observed miRNA profiles clearly distinguished the included sample types (Figure 1): Erythrocytes and leukocytes clustered separately from platelets, while PRP clustered with the platelet samples. The PPP and the supernatant of stimulated platelets formed a separate cluster. The isolation method of platelets (washing steps or column-based) did not substantially alter the miRNA profile, and all of the platelet samples clustered together. All of the abundant platelet miRNAs were also present in other blood cells, suggesting that there were no miRNAs that were exclusively present in platelets. Several thrombomiRs were more abundant or equally abundant in red blood cells and leukocytes as in the platelets. The most abundant miRNAs from the panel were miR-320a, miR-191-5p, and miR-21-5p with average RPMMs (reads per million miRNAs) in platelets of 31,806; 24,686; 19,027. miR-28-3p (4056 RPMMs), miR-27b-3p (1028 RPMMs), and miR-24-3p (2317 RPMMs) also showed high expression in platelets, whereas miR-223-3p (754 RPMMs), miR-126-3p (568 RPMMs), and miR-23a-3p (165 RPMMs) were less abundant. miR-197-3p (42 RPMMs) and miR-150-5p (17 RPMMs) showed very low expression in platelets (Figure S2). miR-150-5p was mainly found in leukocytes (321 RPMMs). We also did not observe higher levels of miR-150-5p in PRP vs. PPP, indicating that platelets are not a major source of this miRNA in the circulation. As controls, we utilized miR-451a (erythrocyte-enriched) and miR-122-5p (liver-specific). As expected, miR-451a was highly abundant in erythrocytes and leukocytes but barely detected in platelets. In line with our expectations, miR-122-5p was only detected in plasma (Figure S2). In general, miRNAs from the pre-selected panel fulfilled our criteria for suitable biomarker candidates of platelet activation, exhibiting the following traits:

1. High abundance in platelets and low abundance in other blood cells;
2. Detectable in PPP;
3. Rise in abundance from PPP to PRP;
4. Detectable in the supernatant of agonist-stimulated platelets.

**Figure 2:**
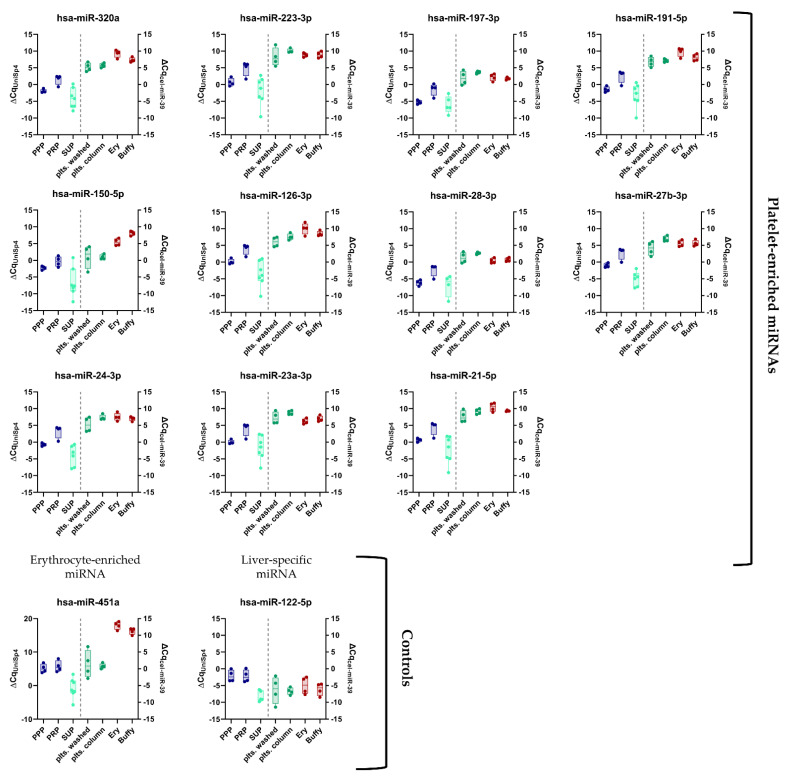
Panel of previously established platelet-enriched miRNAs features promising biomarker candidates of platelet activation. miRNA levels in different blood compartments (red dots on left side = erythrocytes (Ery), red dots on right side = leukocytes (Buffy), and platelets (green dots) (isolation via columns or washing steps), plasma (blue dots = PRP and PPP), and the supernatant of convulxin-stimulated platelets (turquoise dots = SUP)) were determined using RT-qPCR (*n* = 4). Biofluids are depicted on the left, blood cells on the right side of the plots.

Since ligation bias of NGS analysis can lead to over- or underestimation of the endogenous miRNA levels, the NGS results were confirmed using RT-qPCR (Figure 2). For all RT-qPCR experiments, we added synthetic spike-ins in equimolar amounts before each step (i.e., RNA extraction, reverse transcription, and qPCR) to ensure the quality of our results (Figure S8). Indeed, the abundance of miR-451a, miR-223-3p, miR-126-3p, and miR-23a-3p in platelets was underestimated, whereas the abundance of miR-320a and miR-28-3p was overestimated in the NGS analysis (Table S1). In the qPCR analysis, the five most abundant platelet miRNAs from our panel were miR-223-3p, miR-21-5p, miR-23a-3p, miR-191-5p, and miR-126-3p (Table S1). These results show that NGS analysis is suitable for identification of relative differences between similar sample types, while ligation bias limits absolute quantification.

### 3.2. Identification of Novel miRNA-Based Biomarker Candidates of Platelet Activation 

**Figure 3:**
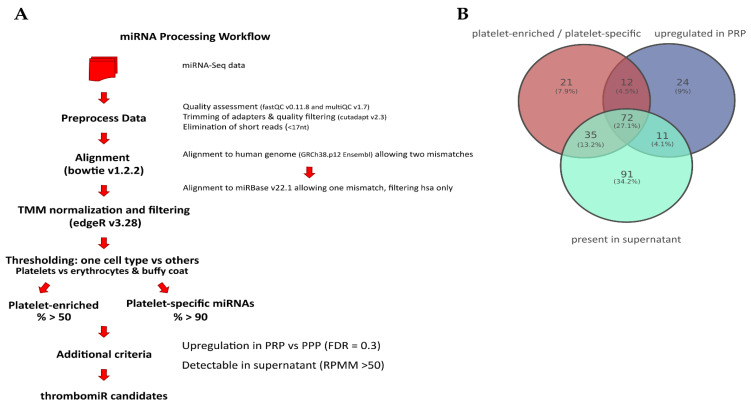

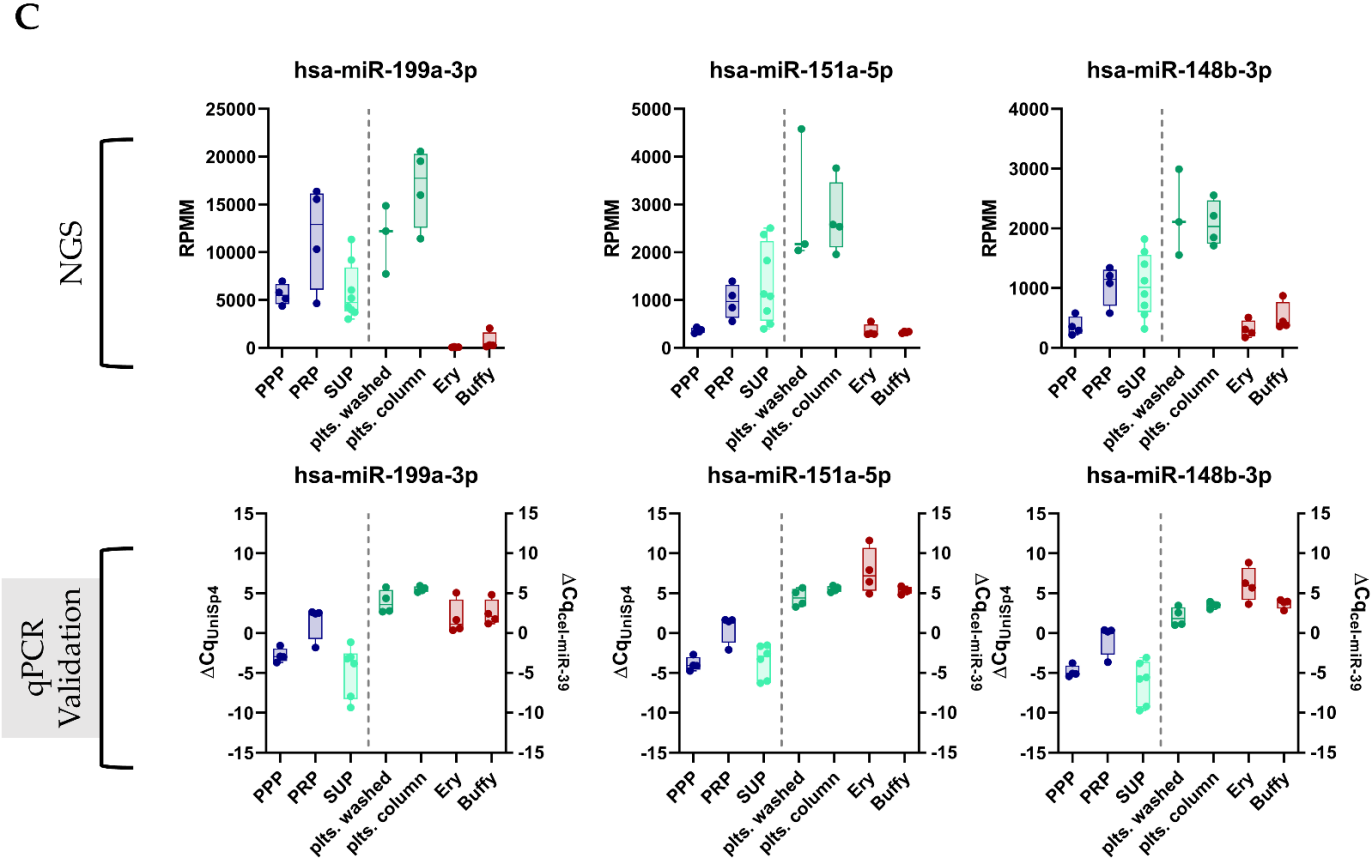
Promising novel biomarker candidates were identified using a self-developed miRNA processing workflow. (**A**) Bioinformatics workflow that was applied to the miRNA-Seq data to identify novel platelet-enriched miRNAs (“thrombomiR candidates”) (adapted from [34]). (**B**) Venn diagram showing that 72 miRNAs fulfilled all of the pre-determined criteria. (**C**) The three most promising novel platelet miRNA candidates represented as RPMMs and ΔCq for qPCR data (*n* = 4). Biofluids are depicted on the left (blue dots = PPP, PRP; turquoise dots = supernatant), blood cells on the right (green dots = platelets; red dots = erythrocytes, leukocytes).

Next, we wondered whether hitherto unexplored miRNAs with improved biomarker qualities could be identified using our NGS data set. To this end, we developed a miRNA processing workflow to select platelet-enriched (PE) and platelet-specific (PS) miRNAs (adapted from [34]). If >50% of the reads of a miRNA were contributed by platelets (and not erythrocytes or leukocytes), this miRNA was considered PE. In case >90% of the reads came from platelets compared to other blood cells, this miRNA was considered PS. In accordance with our previously identified criteria (see Section 3.1), we narrowed down the list of possible biomarker candidates further by selecting PE/PS miRNAs that were also upregulated in PRP vs. PPP (FDR < 0.3) and detectable in the supernatant of activated platelets (RPMM > 50) (Figure 3A). This approach yielded 72 miRNAs (Figure 3B and Figure S3A), five of which were already part of the thrombomiR panel (blue). We selected 21 miRNAs from this list taking into account the detectability in plasma, abundance in other cell types (using the Tissue Atlas [35]), and the added informational content (e.g., excluded closely related mature sequences to already selected miRNAs) and used them for qPCR validation (Figure S3A; grey/yellow). All of the selected platelet miRNAs fulfilled our pre-set criteria, showing the effectiveness of our workflow (Figure S3B). From these 21 newly identified miRNAs, three stood out as exceptionally promising candidates (Figure 3C and Figure S3A; yellow). miR-199a-3p, miR-151a-5p, and miR-148-3p were all highly abundant in platelets with 14,620, 2804, and 2141 RPMMs, respectively. In the qPCR validation, we observed that the abundance of these three miRNAs in platelets was overestimated due to ligation bias (Table S1). Nevertheless, the miRNAs showed excellent measurability in a qPCR setup.

### 3.3. Basal Levels of Circulating Platelet-Enriched miRNAs Are Predominantly Complexed with Proteins, Not Vesicles 

**Figure 4:**
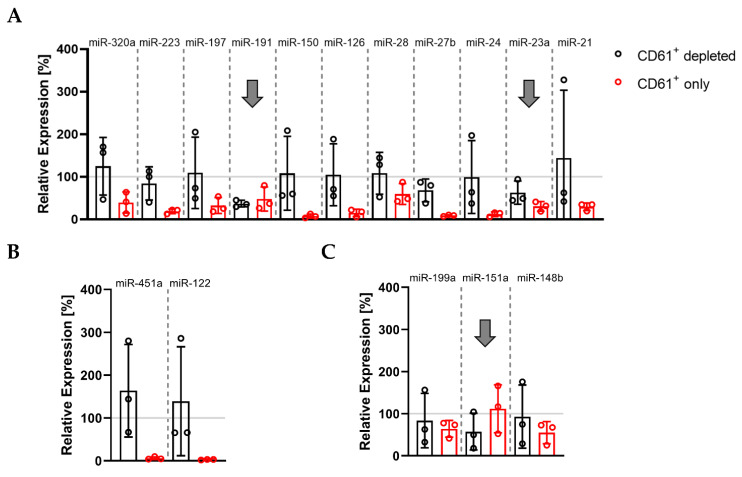
Basal levels of plasma miR-191, miR-151a, and miR-23a circulate in complex with CD61^+^ vesicles: (**A**) miRNAs from the thrombomiR panel; (**B**) control miRNAs; (**C**) novel platelet miRNA candidates. Values represent the relative expression of UniSp4 normalized Cq values that were linearized (ΔΔCq; % of control samples (PPP)). Arrows mark miRNAs with an only fraction > 30% and a depleted fraction < 70% (average of three independent donors).

We depleted and enriched MVs with different cellular origins in PPP. This allowed us to explore the abundance of thrombomiRs in MVs derived from platelets (CD61^+^) and other blood cell types. The pattern we were looking for was a decrease in the relative expression of the CD61^+^-depleted fraction to <70% and a rise in the CD61^+^-only fraction to >30% in comparison to the total miRNA amount in PPP (average of three biological replicates). These criteria were met by three miRNAs from our panel: miR-191-5p, miR-151a-5p, and miR-23a-3p, indicating that these miRNAs circulate within platelet-derived vesicles (Figure 4A–C). The other platelet-enriched miRNAs were thus likely to be predominantly complexed with proteins rather than vesicles. The control miRNAs, miR-451a and miR-122-5p, were protein-bound. In addition, the presence of the selected miRNA panel was analyzed in CD45^+^, CD235b^+^, and CD31^+^ vesicles. We identified several of our miRNA candidates as present in CD235b^+^ vesicles, indicating a potential erythrocyte origin in plasma: miR-223-3p, miR-199a-3p, miR-197-3p, miR-191-5p, miR-151a-5p, and miR-23a-3p (Figure S4).

### 3.4. Alterations in Plasma miRNA Abundance due to Platelet Activation and Subsequent miRNA Secretion Are Detectable with qPCR

**Figure 5:**
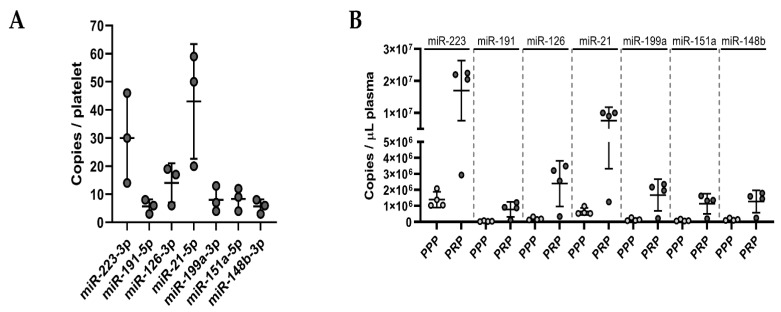

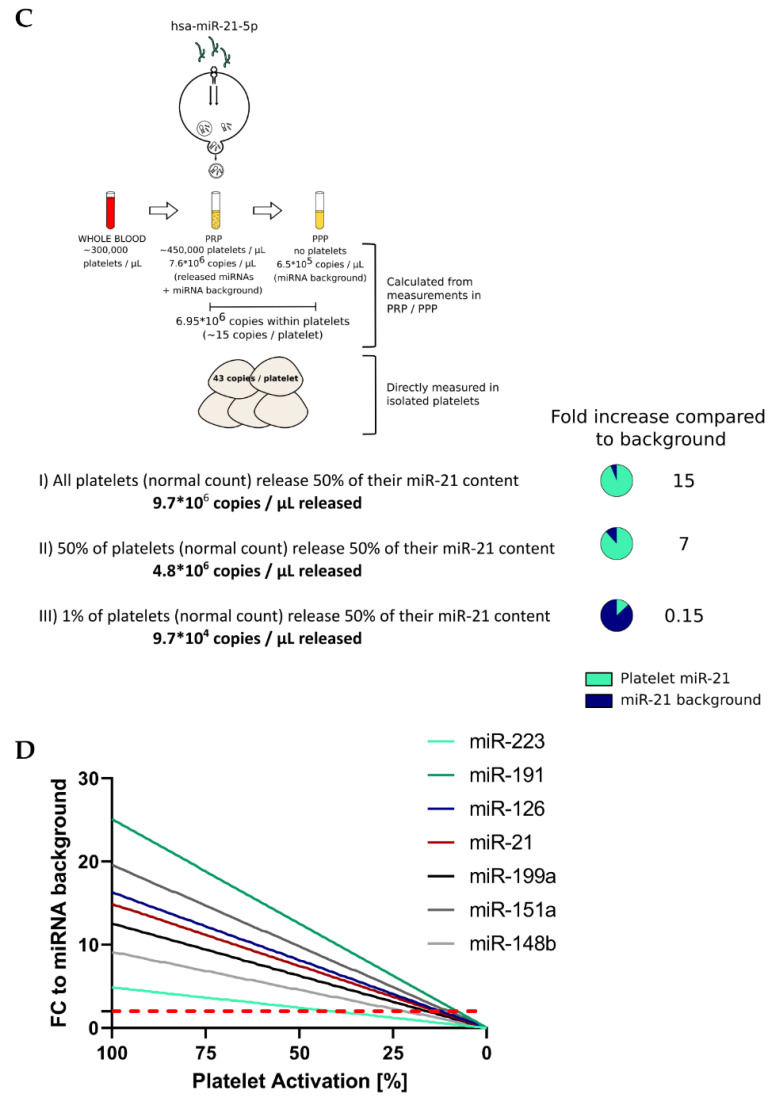
Ex vivo release of miRNAs due to platelet activation is detectable with qPCR. (**A**) Absolute quantification of a panel of seven miRNAs in platelet pellets (known number of cells; *n* = 3). Copies/platelet were calculated using the standard curve method. (**B**) Absolute quantification of seven platelet miRNAs in PRP and PPP (*n* = 4). Copies/μL plasma were determined using standard curves. (**C**) Assuming three different simulation scenarios, we calculated the theoretical release of miRNAs by activated platelets and compared it to the miRNA background to assess the detectability of such miRNA alterations. (**D**) The percentage of activated platelets from 100–0% (assuming 50% miRNA release upon activation) was plotted against the corresponding alterations in miRNA levels (compared to the background) in PPP. The dotted, red line represents the FC threshold (2) above which alterations of miRNA levels would be detectable in an RT-qPCR setup.

Naturally, the miRNA background in plasma is high due to the release of miRNAs from a variety of cell types. MiRNAs from our panel are not exclusive to platelets; therefore, other cells contribute to the basal levels of these miRNAs in the circulation. We were interested in whether the ex vivo release of miRNAs induced by platelet activation could lead to a measurable rise in thrombomiR levels in PPP (exceeding the miRNA background). To answer this question, we performed absolute miRNA quantification in platelets (Figure 5A), PRP, and PPP (Figure 5B and Figure S5). We chose four miRNAs from the established panel and the three most promising novel candidates. This approach enabled us to calculate copies/platelet, copies/μL PRP, and copies/μL PPP for each miRNA (Table 4). The most abundant miRNA in platelets was miR-21-5p (43 copies/platelet), followed by miR-223-3p (30 copies/platelet), and miR-126-3p (14 copies/platelet). The remaining miRNAs had an average copy number of <10 per platelet (Figure 5A). Complementary to this direct analysis of the miRNA copy numbers in platelets, we tried to confirm these results by calculating the miRNA copy number in platelets using the measurements of PPP and PRP samples from healthy subjects with available platelet counts (Figure 5C and Table 4). The calculated and the directly measured copy numbers per platelet matched well, emphasizing the robustness of the approach. We observed that miR-223-3p was the most abundant platelet miRNA (35 copies/platelet), followed by miR-21-5p (15 copies/platelet) (Table 4).

After establishing microRNA copy numbers in platelets, we were interested in whether the miRNA release from platelets after activation could be high enough to result in measurable increases (according to the fold change (FC)) relative to baseline (background) levels in PPP. Therefore, we determined miRNA copy numbers in PPP and found that miR-223-3p and miR-21-5p had the highest miRNA background in PPP (1.4 × 10^6^ and 6.5 × 10^5^ copies/μL PPP). Finally, we used the generated data to simulate three scenarios (Figure 5C and Table 4):

1. All platelets in PRP (from a healthy donor with a normal platelet count) release 50% of their miRNA content upon agonist stimulation (“best-case” of expected miRNA rise in plasma);
2. Half of the platelets present in PRP (from a healthy donor with a normal platelet count) secrete half of their miRNA content (“middle-case”);
3. One percent of the platelets in PRP (from a healthy donor with a normal platelet count) release 50% of their miRNA content upon agonist stimulation (“worst-case”).

For all seven miRNAs from the panel, the miRNA release would be detectable in scenarios I and II but not in the worst-case scenario. For miR-21-5p, we would expect a 15-fold increase compared to the background and a seven-fold increase for scenario II still. Both cases would be clearly detectable in an RT-qPCR setup. Only in scenario III, the miRNA background would mask the minute release. When presupposing that 50% of a miRNA species is released from platelets upon activation, almost all scenarios (0–100% of platelets activated) lead to a detectable rise (FC ≥ 2) of the respective miRNA above the baseline in plasma (Figure 5D). These data encouraged us to take a closer look at the in vitro release kinetics of platelet miRNAs.

### 3.5. Platelets Release miRNAs upon Agonist Stimulation in PRP 

**Figure 6:**
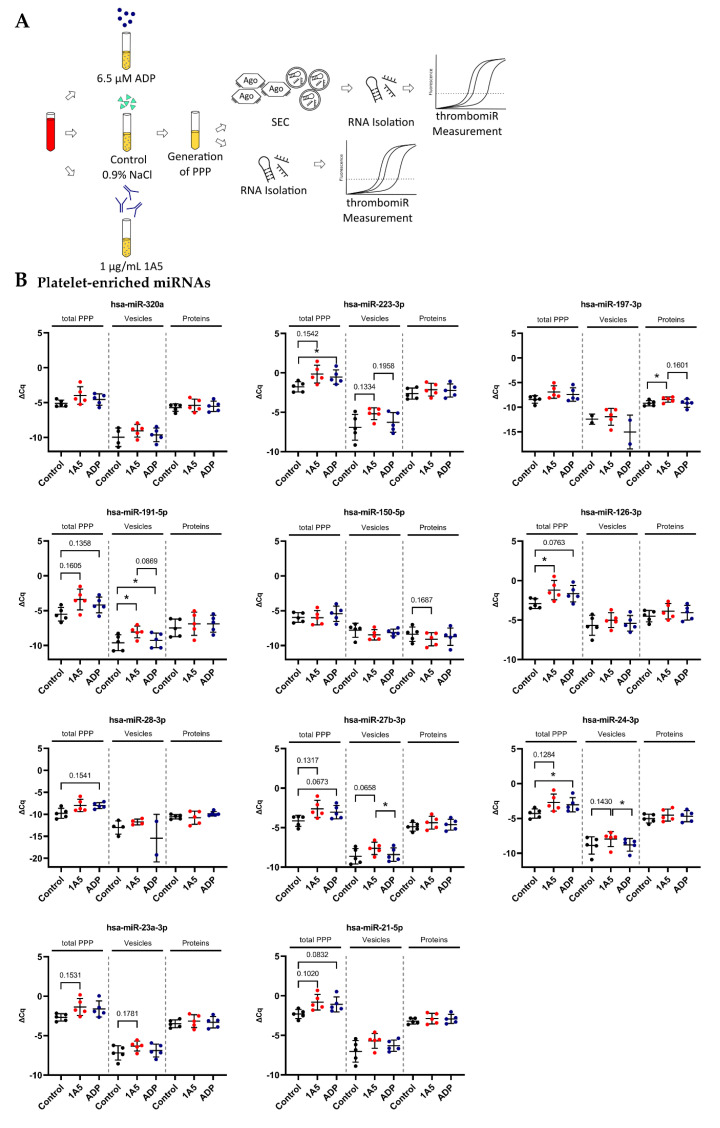

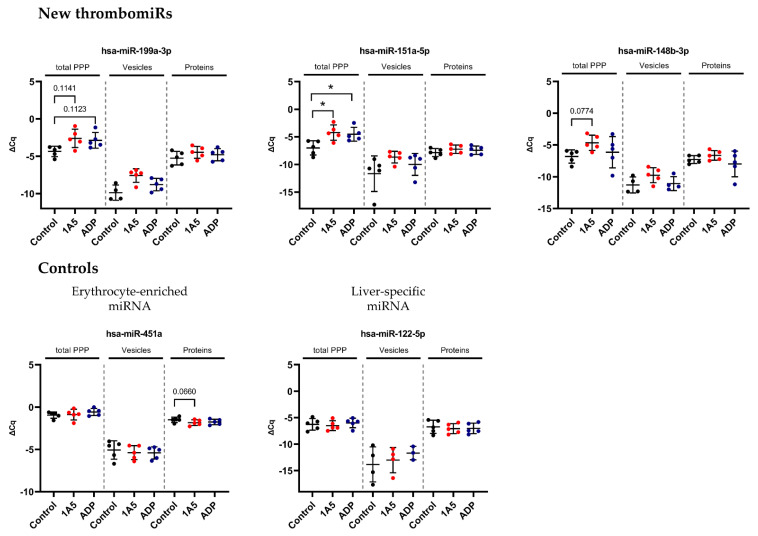
In vitro activation of platelets in plasma results in detectable secretion of miRNAs. (**A**) Experimental setup of in vitro experiments performed to investigate the release of miRNAs upon platelet activation in PRP. Two different agonists were used: 6.5 μM ADP and 1 μg/mL 1A5. Addition of an equal volume of 0.9% NaCl served as unstimulated control. PPP samples were used directly for RNA isolation (total PPP) or as input for SEC. (**B**) miRNA levels in total PPP and the vesicle and protein fractions (*n* = 5). qPCR data were RNA spike-in normalized. Black dots indicate control samples, red dots indicate stimulation with 1A5, and blue dots signify platelet activation using ADP. The mean ± SD are plotted. Repeated-measures one-way ANOVA with Sidak’s multiple comparison test was calculated. In case of missing values, a mixed-effects analysis was performed. *p*-Values < 0.05 were considered significant. *p*-Values between 0.05 and 0.2 are depicted. * = *p* ≤ 0.05.

To verify the calculations and estimations in Section 3.4, we stimulated platelets in PRP from five healthy donors using 1A5 (GPVI receptor agonist) and ADP. To ensure that platelets were actually activated, we measured P-selectin (CD62P) expression as well as platelet aggregation. Platelet stimulation with 1A5 and ADP led to upregulation of CD62P expression as well as platelet aggregation in comparison to the untreated control samples (Figure S7). After generation of PPP, samples were divided and used for RNA isolation directly (total PPP) or size-exclusion chromatography (SEC) to separate vesicle- and protein-bound miRNAs (Figure 6A). Almost all miRNAs from our panel were increased in the stimulated samples. We observed a significant (*p* < 0.05) rise upon ADP stimulation (in total PPP) of miR-223-3p, miR-151a-5p, and miR-24-3p as well as a clear trend (*p* < 0.2) towards upregulation for miR-199a-3p, miR-191-5p, miR-126-3p, miR-28-3p, miR-27b-3p, and miR-21-5p. Stimulation of platelets with 1A5 resulted in a significant rise in total PPP of miR-151a-5p and miR-126-3p as well as a trend towards increased levels for miR-223-3p, miR-199a-3p, miR-191-5p, miR-148b-3p, miR-27b-3p, miR-24-3p, miR-23a-3p, and miR-21-5p. Several analyzed miRNAs were more abundant in the protein compared to the EV fraction. However, we also identified miRNAs, such as miR-191-5p, to be equally present in vesicles and protein complexes. Interestingly, vesicle-bound miR-191-5p was significantly increased in the stimulated samples. We also observed a trend towards upregulation of vesicle-bound miR-223-3p, miR-27b-3p, miR-24-3p, and miR-23a-3p upon 1A5 stimulation of platelets. From the protein fraction, only miR-197-3p showed a significant increase upon 1A5 treatment and a trend towards upregulation upon ADP stimulation. Liver-specific miR-122-5p and erythrocyte-enriched miR-451a were not affected by platelet stimulation (Figure 6B).

### 3.6. Isolated Platelets Secrete miRNAs upon Activation in Buffer

**Figure 7:**
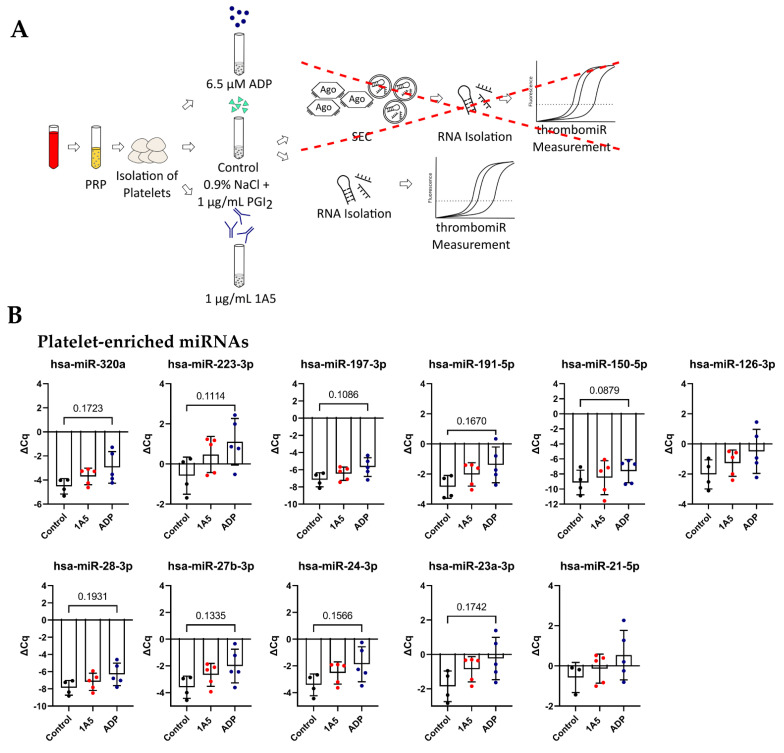

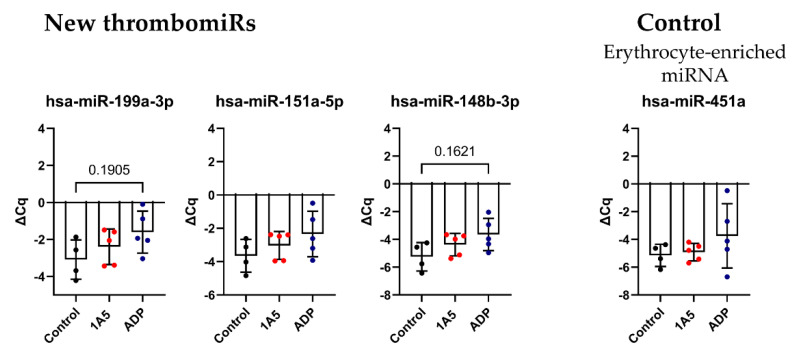
Activation of isolated platelets in buffer results in miRNA release. (**A**) Experimental setup to investigate the release of miRNAs upon platelet activation in buffer. Two different agonists were used: 6.5 μM ADP and 1 μg/mL 1A5. Addition of 1 μg/mL PGI_2_ served as negative control. Supernatants were used directly for RNA isolation. (**B**) miRNA levels in the supernatant of activated platelets. qPCR data were RNA spike-in normalized (*n* = 5). The mean ± SD are plotted. We performed a mixed-effects analysis with Tukey’s multiple comparisons test. *p*-Values < 0.05 were considered significant. *p*-Values between 0.05 and 0.2 are depicted.

In order to reduce the “background” and verify these results, we isolated and stimulated (ADP (6.5 μM) and 1A5 (1 μg/mL)) platelets in buffer, followed by the collection of supernatant. P-selectin (CD62P) expression and platelet aggregation increased upon agonist treatment (Figure S7). In this context, enrichment of EVs (extracellular vesicles) by SEC was not performed, since we observed that the amount of RNA that could be recovered was below the limit of detection of our assay. Thus, we isolated RNA only from the total supernatant and did not distinguish between vesicle- and protein-bound miRNAs (Figure 7A). Surprisingly, with this setup, the difference between the unstimulated control and the stimulated samples was smaller. Still, a clear trend towards upregulation upon ADP stimulation was visible for all miRNAs from our panel, except miR-151a-5p, miR-126-3p, and miR-21-5p. As before, miR-451 was not affected by agonist stimulation (Figure 7B), and liver-specific miR-122-5p was not detected, as there were only miRNAs present that were released from isolated platelets. The miRNA background in the controls was unexpectedly high. To further investigate this observation, we compared our previously used control to maximally inhibited controls (extra addition of 1 μg/mL PGI_2_ during the incubation step and reduction of centrifugation speed). A clear trend towards lower levels in the maximally inhibited control was clearly visible for most measured miRNAs, almost reaching significance in miR-191-5p, miR-151a-5p, miR-126-3p, and miR-24-3p. This experiment further demonstrated that generating adequate unstimulated controls without artificial miRNA release from platelets is technically demanding (perhaps impossible) (Figure S6A). The problem of pre-analytical variability in this setup was further exacerbated by rather high variability between replicates for platelet miRNAs (median ΔΔCq in total PPP = 0.92) but not miRNAs predominantly derived from other sources (miR-451a, miR-122-5p, and miR-150-5p) (median ΔΔCq in total PPP = 0.29). We detected a difference of ~0.5–1 Cq between technical replicates (Figure S6B). 

### 3.7. In Vivo Thrombosis Results in the Release of Platelet miRNAs in Mice

**Figure 8:**
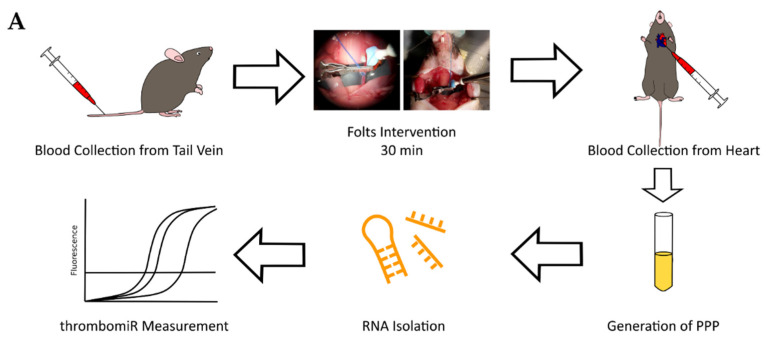

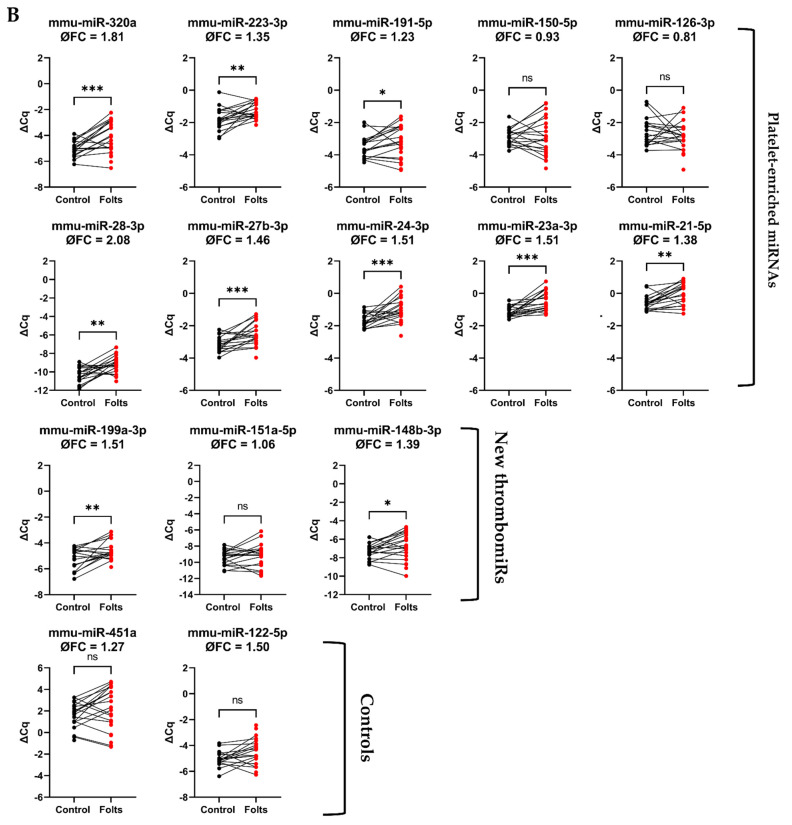
Platelet-enriched miRNAs are released upon in vivo thrombosis. (**A**) Experimental setup to study in vivo thrombogenesis in mice. (**B**) miRNA levels in PPP of mice before (black dots) and after the Folts intervention (red dots) (*n* = 25). qPCR data were RNA spike-in normalized. We performed a paired *t*-test. *p*-Values < 0.05 were considered significant. * = *p* ≤ 0.05, ** = *p* ≤ 0.01, *** = *p* ≤ 0.001.

Following the in vitro experiments, we set out to confirm our findings in vivo. To this end, we measured thrombomiR levels in PPP of 25 mice undergoing the Folts procedure (Figure 8A). The Folts intervention is a surgical procedure in which the carotid artery in the neck is injured and compressed to trigger recurring thrombus formation and dislodging. Before the intervention, blood was collected from the tail vein (baseline). After 30 min of recurring CFRs, blood was drawn from the heart. The method of phlebotomy (tail vein vs. heart) did not significantly affect circulatory miRNA levels (Figure S9). Using this mouse model, we observed a strong and significant release of thrombomiRs from our panel. Only miR-150-5p, miR-126-3p, and miR-151-5p (new thrombomiR) showed no significant effect. miR-197-3p does not have a mouse ortholog and could therefore not be measured in this context. The control miRNAs (i.e., miR-451a and miR-122-5p) did not significantly increase as a result of the Folts intervention (Figure 8B).

## 4. Discussion

The main aim of this work was to explore the utility of platelet-derived miRNAs as in vitro and in vivo surrogate markers of platelet activation. Based on the considerable body of knowledge gathered by previous studies, we hypothesized that certain platelet miRNAs are secreted upon platelet activation, resulting in detectable alterations of plasma miRNA levels. First, we confirmed a panel of previously suggested miRNA biomarker candidates in an unbiased small RNA-Seq-based approach. As controls, we utilized miR-451a, known to be enriched in erythrocytes [36] and miR-122-5p, a liver-specific miRNA [37,38]. None of the abundant platelet miRNAs were exclusive to this cell type. In fact, many miRNAs from the panel are also abundant in erythrocytes and leukocytes. For example, miR-150 was mainly present in leukocytes, an observation that is in line with previous reports [39]. Respective biomarker candidates might therefore also be released from other blood compartments into the circulation, not only platelets. This finding is not unexpected, as there are very few miRNAs exhibiting tissue specificity (such as liver miR-122). Still, platelets are known to strongly contribute to circulatory miRNA levels [11,12] and cells with higher miRNA content than platelets can still contribute less to the pool of cell-free miRNAs in the blood. When working with NGS, ligation bias leads to over- or underestimation of miRNA levels and cannot be entirely bypassed. Several miRNAs from our panel of interest were affected by ligation bias, illustrating the importance of validating the results in an RT-qPCR setup. However, by combining our NGS-based workflow with RT-qPCR validation, we were able to confirm our existing panel of platelet miRNAs (thrombomiR) as well as select promising new miRNA biomarker candidates. We adapted a previously described workflow for identification of tissue-enriched miRNAs [34] and deliberately used broad criteria to not exclude interesting candidates. This allowed us to identify 72 potentially platelet-enriched miRNAs, of which five miRNAs were already part of our panel, substantiating the validity of our bioinformatics analysis. In a second step, we were more stringent and chose 21 miRNAs to validate in an RT-qPCR setup. In this step, we were mainly interested in whether the chosen candidate miRNAs were robustly detectable in plasma, as this is a common restraint of this type of analysis. Further criteria included the abundance of the respective miRNA in other cell types and the added informational content (closely related sequences to other miRNAs in the panel were removed). Ultimately, we picked three candidates, miR-199a-3p, miR-151a-5p, and miR-148b-3p, that stood out as exceptional.

Enquiry of the current literature exposes some controversy regarding the predominant carrier of cell-free miRNAs circulating in the blood. Many studies report that circulatory miRNAs are bound to (Argonaute) proteins, protecting them from degradation in this harsh environment [40,41]. On the other hand, activated platelets have been described to release so called platelet-derived MVs (PdMVs) [42,43], indicating that miRNAs secreted during this process might be vesicle-bound. Our data support the notion that basal levels of platelet-derived miRNAs in the circulation are predominantly protein-bound. Only three miRNAs (i.e., miR-191-5p, miR-151a-5p, and miR-23a-3p) were detected in CD61^+^ (platelet-derived) vesicles. The control miRNAs miR-451a (red blood cell-enriched) and miR-122-5p (liver-specific) were not found in complex with vesicles, as expected [40]. The variability between different donors was relatively high for some miRNAs, impeding interpretation of the results. Nevertheless, the variability between donors was not especially high in the miRNAs we identified as circulating in complex with platelet-derived EVs. Moreover, we observed that miRNAs from our panel were not detected in the EV fraction but in the total RNA fraction of platelets stimulated in buffer. This finding suggests that activated platelets not only secrete miRNAs within EVs but also bound to proteins. One previous study found no Argonaute proteins in the protein pool secreted from activated platelets [44]; however, we speculate that this is due to lack of sensitivity of the applied mass-spectrometry method as well as low levels of Argonaute proteins released. Considering the high miRNA background in human plasma, we were curious whether the release of miRNAs from platelets could be detected using RT-qPCR. To this end, we devised a simulation with three scenarios (i.e., best-case, middle-case, and worst-case) supported by empirical data. In the best-case scenario, all platelets release half of their miRNA content upon agonist stimulation. The middle-case illustrates a situation in which half of the platelets secrete half of their miRNA content, whereas in the worst-case, 1% of platelets release 50% of their miRNA content. We assumed a fixed volume of plasma, a normal platelet count, and impermeable platelets. In scenarios I and II (i.e., best- and middle-case), we calculated a rise of plasma miRNA abundance compared to the miRNA background upon activation of 5–25-fold and 2–13-fold, respectively. Both cases would be clearly detectable in a qPCR setup. In the worst-case scenario, the miRNA background exceeded the release. It is conceivable that high levels of platelet activation in patients suffering from CVDs as well as the efficacy of drug-induced platelet inhibition can be detected by miRNA analysis. Undoubtedly, these insights cannot be transferred to an in vivo setting without further investigations, as there is:

1. No fixed blood volume at a certain location;
2. Only a subpopulation of platelets is activated (in case of CVDs);
3. Presumably not the entire miRNA content of one species is secreted per platelet.

Still, we considered the continuance of our experiments warranted. We conducted a series of in vitro experiments, studying the miRNA release upon agonist stimulation of platelets in PRP or buffer. Using an optimized protocol to achieve the lowest possible degree of artificial miRNA release, we clearly observed the secretion of miRNAs upon platelet activation, even reaching significance for several biomarker candidates from our panel. In a more artificial setting (platelet extraction and agonist stimulation in buffer), the miRNA background in the unstimulated controls was unexpectedly high. Nevertheless, we found the same trend towards upregulation of platelet-derived miRNAs in the stimulated samples. Previous studies show that miR-126 is released from activated platelets, whereas platelet inhibition attenuates the secretion [3]. Additionally, elevated levels of circulatory miR-126 were linked to an increased risk of major adverse cardiac events (MACE) and miR-126 levels were correlated with markers of platelet activation [3]. These findings indicate that circulating miR-126 levels might be primarily derived from platelets (and not endothelial cells as previously reported) [3]. In our study, we found that basal levels of miR-126 were not complexed with platelet-derived EVs. We showed that activated platelets secrete miR-126; however, due to limitations of in vitro experiments with platelets, our experiments did not allow us to definitely establish the main carrier of this miRNA in the circulation. A previous publication reports that agonist stimulation of platelets in vitro results in the release of approximately 50 different miRNA species [13], but the authors do not provide data on miRNA levels in unstimulated controls. Hence, the observed effects could also be the result of sample handling. In both of our experimental setups, effect sizes remained rather small due to low sample numbers and the artificial release of miRNAs during sample handling. In vitro experiments with platelets remain technically challenging, as platelets constantly release cell fragments (also containing miRNAs) ex vivo, especially during centrifugation (“platelet blebbing”), overshadowing the miRNA release induced by agonist treatment. This fact renders the generation of adequate unstimulated controls without artificial miRNA release practically impossible. Our experiments were further complicated by rather high pre-analytical variability, sometimes exceeding the target biological variability. We determined around 0.5–1 Cq difference between technical replicates. Interestingly, the variability was strongly reduced in miRNAs without predominant platelet origin (miR-451a, miR-122, and miR-150) (median ΔΔCq in total PPP = 0.92 (platelet miRNAs) vs. 0.29 (platelets not main source)), without those miRNAs being more abundant than the platelet miRNAs.

With this article, we also aim to raise awareness that studying the release of molecules from platelets in vitro is a delicate matter. Standardized protocols and careful sample handling are central in this context [45,46]. Removing residual platelets by double centrifugation or prolonged single centrifugation to gain PPP before freezing the sample is important to consider, as an omission cannot be corrected subsequently. Freezing and thawing of plasma samples with residual platelets leads to artificial miRNA release [47]. The degree of cellular contamination of plasma samples also depends on pipetting routines upon aspirating PRP layer or pellet supernatant, causing variable residual platelet counts and biasing analyses [48]. All of this demonstrates that many intricacies have to be considered to receive meaningful results.

To further corroborate our findings in an in vivo setting, we performed thrombomiR measurements in the plasma of a mouse model of in vivo thrombosis. Upon recurring thrombus formation, we observed a strong and statistically significant release of thrombomiRs. Interestingly, miRNAs predominantly derived from other cell sources (i.e., miR-150-5p, miR-126-3p, miR-451a, and miR-122-5p) were not significantly altered upon recurring thrombus formation. This supports our hypothesis that the observed effect was caused by miRNA release from platelets and is not systemic. In regard to miR-126, we did not expect this result, as it has been speculated before that activated platelets might be the main contributor to circulating levels of this miRNA [3]. As mentioned above, elevated levels of circulatory miR-126 have also been associated with the occurrence of MACE [3]. In accordance with these previous reports, we expected a rise in plasma miR-126 levels in mice undergoing the Folts intervention. Nevertheless, only the three miRNAs from our panel with major additional cellular sources (i.e., miR-451, miR-150, and miR-126) showed a significant effect upon thrombosis. Consequently, we speculate that activated platelets might not be the primary source of plasma miR-126 (in mice). In general, the predominant cellular origin of many blood-based miRNAs is currently unexplored/controversial. It is also conceivable that differences in mouse and human platelets might contribute to the unexpected result. miR-122-5p was not significantly increased; however, there was a trend towards upregulation, indicating that this miRNA might not be an ideal control in this setting as surgery/poor general health can be linked to increased plasma miR-122 levels. Of note, there were several animals in which thrombomiR levels were decreased after the intervention. We suspect that this might be due to large thrombi trapping miRNAs. 

With this work, we provide a comprehensive and unbiased approach to confirm/identify miRNA biomarker candidates of platelet activation, determine their predominant carrier in the circulation, and show their utility in an in vitro as well as in vivo setup. Open questions remain such as additional cellular origins, the influence of comorbidities/medications, or whether miRNAs are released in bulk or selectively. Follow-up clinical studies will elucidate whether the miRNA panel would benefit from including the three novel thrombomiRs or whether the entire panel can be scaled down to reduce redundancies of information and increase throughput. However, our results warrant further investigations, especially examining the utility of the thrombomiR panel in patient cohorts or settings in which volunteers receive antiplatelet treatments to corroborate previous studies and correlate platelet-derived miRNA levels to PFTs. Given the evidence we have collected, a link between platelet reactivity and the circulating levels of platelet-derived miRNAs seems plausible.

## Figures and Tables

**Table 1:** Summary of experiments and experimental parameters.

Experiment	Aim	Biological Replicates	Anticoagulant	Input RNA Extraction	RNA Input RT/Library Preparation	Agonist
I (Figure 1, Figure 2 and Figure 3)	NGS analysis of blood cells, plasma, and supernatant of stimulated platelets	4	CTAD	Plasma: 170 μL	Cells: 10 ng Plasma: 2 μL	Convulxin: 25 ng/mL
II (Figure 4)	Microvesicle enrichment and depletion	3	CTAD	80 μL	2 μL	-
III (Figure 5)	Absolute quantification of platelet miRNAs	3/4	Citrate	PRP/PPP: 170 μL	2 μL	-
IV (Figure 6 and Figure 7)	In vitro activation of platelets	5	Citrate	SEC: 150 μL PPP Total PPP: 50 μL Supernatant: 200 μL	2 μL	Control: 0.9% NaCl (+1 μg/mL PGI_2_) 1A5: 1 μg/mL ADP: 6.5 μM 500× *g* centrifugation during wash steps
V (Figure S6A)	Study the platelet miRNA background introduced by sample handling	5	Citrate	200 μL supernatant	2 μL	Control + PGI_2_: 0.9% NaCl + 1 μg/mL PGI_2_ Control: 0.9% NaCl
VI (Figure S6B)	Study the technical variability of the in vitro activation workflow	5 (two technical replicates/donor)	Citrate	SEC: 150 μL Total PPP: residual volume (~40 μL; calculated back to equal input for all samples)	2 μL	Control: 0.9% NaCl 1A5: 1 μg/mL ADP: 6.5 μM
VII (Figure 8)	Study the effect of the Folts intervention on circulatory miRNA levels	25	Citrate	20 μL PPP	2 μL	-
VIII (Figure S9)	Study the effect of the blood withdrawal method on plasma miRNA levels in mice	3	Citrate	20 μL PPP	2 μL	-

**Table 2:** miRNA mimic sequences used for the standard curves.

miRNA ID	Sequence
hsa-miR-223-3p	UGUCAGUUUGUCAAAUACCCCA
hsa-miR-199a-3p	ACAGUAGUCUGCACAUUGGUUA
hsa-miR-191-5p	CAACGGAAUCCCAAAAGCAGCUG
hsa-miR-151a-5p	UCGAGGAGCUCACAGUCUAGU
hsa-miR-148b-3p	UCAGUGCAUCACAGAACUUUGU
hsa-miR-126-3p	UCGUACCGUGAGUAAUAAUGCG
hsa-miR-21-5p	UAGCUUAUCAGACUGAUGUUGA

**Table 3:** ThrombomiR panel.

miRNA	Abundance in Platelets	Platelet Contribution to Plasma Levels	Suitability as (Platelet) Biomarker (Inter Alia)	Sources	Reference
miR-320a	**	***	Potential biomarker for arrhythmogenic cardiomyopathy	Platelets, endothelial cells, and cardiomyocytes	[27]
miR-223-3p	****	****	Reduced plasma levels in healthy donors and CVD patients in response to antiplatelet therapy	Platelets	[11]
miR-197-3p	*	***	Potential biomarker of CVD in patients with T2DM	Platelets	[28]
miR-191-5p	***	****	Reduced plasma levels in healthy donors and CVD patients in response to antiplatelet therapy	Platelets and endothelial cells	[11]
miR-150-5p	*	*	Reduced plasma levels in healthy donors and CVD patients in response to antiplatelet therapy	Platelets and leukocytes	[11]
miR-126-3p	***	***	Linked to occurrence of MI in the general population	Platelets and endothelial cells	[29]
miR-28-3p	*	***	Potential biomarker of pulmonary embolism	Platelets and cardiomyocytes	[30]
miR-27b-3p	**	***	Potential biomarker of severe atherosclerosis	Platelets and endothelial cells	[31]
miR-24-3p	***	***	Reduced plasma levels upon potent platelet inhibition in patients with T2DM	Platelets, endothelial cells, and vascular smooth muscle cells	[28]
miR-23a-3p	***	***	Potential biomarker of coronary artery disease	Platelets	[32]
miR-21-5p	***	***	Elevated plasma levels in patients with AMI and angina pectoris	Platelets, vascular smooth muscle cells, endothelial cells, and cardiomyocytes	[33]
miR-451a	***	-	-	Erythrocytes	-
miR-122-5p	-	-	-	Liver cells	-

The average abundance of the selected miRNAs in platelets was calculated using the qPCR data (Figure 2). The platelet contribution of the individual thrombomiRs to plasma levels was estimated based on the average rise from PPP to PRP (qPCR data, Figure 2). **** Very high, *** high, ** low, * very low, and - not detected/no platelet contribution.

**Table 4:** Ex Vivo Release of Seven ThrombomiRs due to Platelet Activation (* Copies/μL PRP—Copies/μL PPP)/450,000).

	miR-223	miR-191	miR-126	miR-21	miR-199a	miR-151a	miR-148b
Copies/platelet (average of *n* = 3)	30	5	14	43	8	8	6
Copies/μL PPP (average of *n* = 4)	1.4 × 10^6^	4.5 × 10^4^	1.9 × 10^5^	6.5 × 10^5^	1.4 × 10^5^	9.2 × 10^4^	1.5 × 10^5^
Copies/μL PRP (average of *n* = 4)	1.7 × 10^7^	7.8 × 10^5^	2.4 × 10^6^	7.6 × 10^6^	1.7 × 10^6^	1.1 × 10^6^	1.3 × 10^6^
Copies/platelet (calculated) *	35	2	5	15	3	2	3
(I) 100% of platelets release 50% of their miRNAs in PRP (copies/μL)	6.8 × 10^6^	1.1 × 10^6^	3.2 × 10^6^	9.7 × 10^6^	1.8 × 10^6^	1.8 × 10^6^	1.4 × 10^6^
(II) 50% of platelets release 50% of their miRNA content (copies/μL)	3.4 × 10^6^	5.6 × 10^5^	1.6 × 10^6^	4.8 × 10^6^	9.0 × 10^5^	9.0 × 10^5^	6.8 × 10^5^
(III) 1% of platelets release 50% of their miRNA content (copies/μL)	6.8 × 10^4^	1.1 × 10^4^	3.2 × 10^4^	9.7 × 10^4^	1.8 × 10^4^	1.8 × 10^4^	1.4 × 10^4^
**Fold Increase Compared to Baseline**							
(I)	5	25	16	15	13	20	9
(II)	2	13	8	7	6	10	5
(III)	0.05	0.25	0.16	0.15	0.13	0.20	0.09

## Data Availability

The sequencing data are available in the Gene Expression Omnibus repository (GSE200251).

## Supplementary Materials

The following are available online at https://www.mdpi.com/article/10.3390/cells11081254/s1: Figure S1: Reads classification and miRNA mappings; Figure S2: miRNA levels in blood cells and plasma from four healthy volunteers; Table S1: Comparison of qPCR and NGS data; Figure S3: Validation of novel thrombomiR candidates; Figure S4: Basal levels of circulatory thrombomiRs in all measured vesicle fractions; Figure S5: Standard curves and lines fitted to the data of the dilution series of each miRNA; Figure S6: In vitro detection of miRNA secretion upon platelet activation remains challenging; Figure S7: Platelet activation and aggregation markers in the samples of the in vitro experiments; Figure S8: Quality control of all samples; Figure S9: The method of blood withdrawal from mice (tail vein vs. heart) does not significantly affect thrombomiR levels in PPP.
