# Supplementary material for: Comprehensive Characterization of Platelet-Enriched MicroRNAs as Biomarkers of Platelet Activation
Source: Cells. 2022 Apr 7;11(8):1254. doi: 10.3390/cells11081254 (PMC9030873; doi:10.3390/cells11081254)
Supplement: Supplementary file 1 [file cells-11-01254-s001.zip › cells-1608581-supplementary.pdf]

Supplementary Results

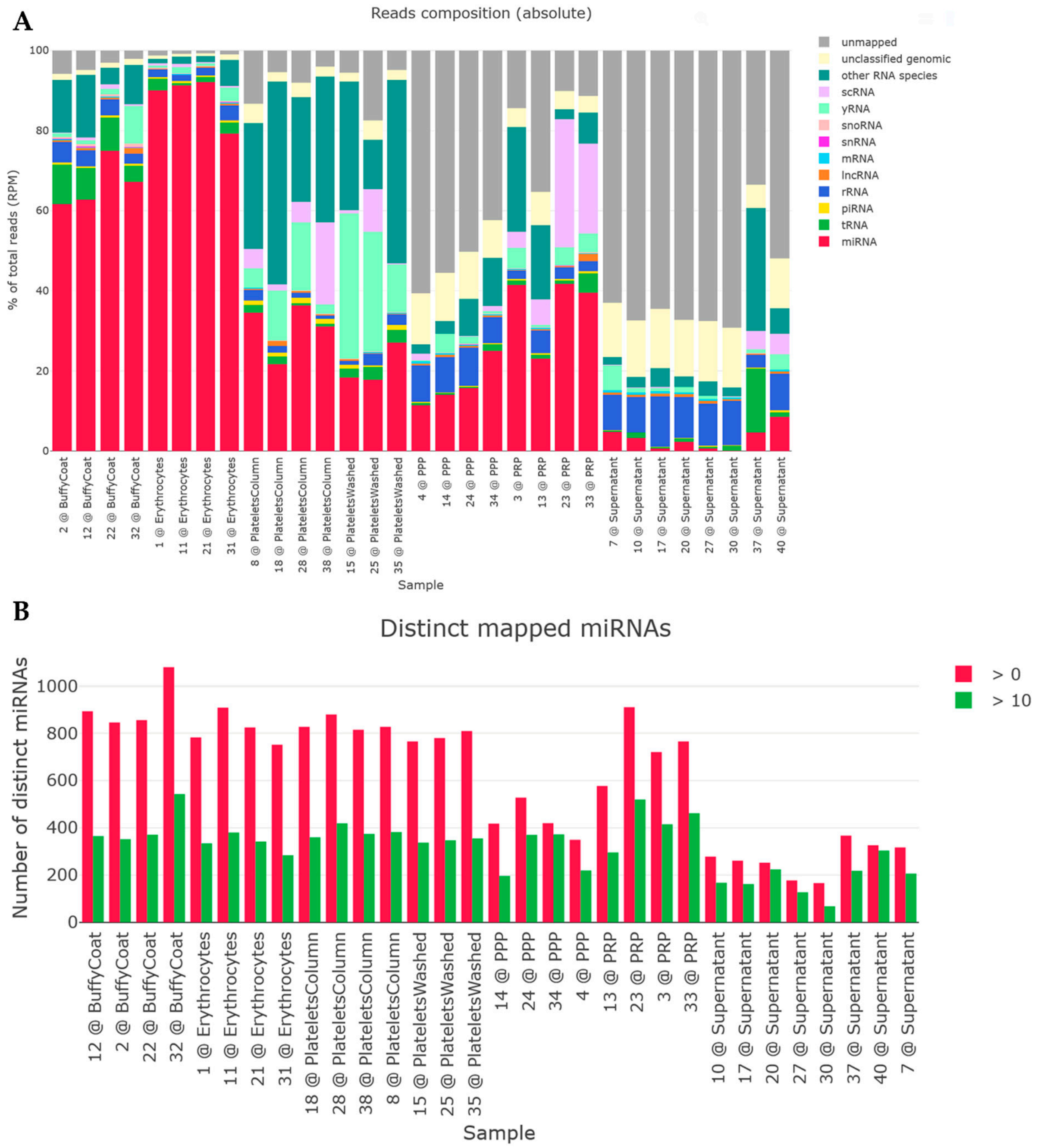

Figure S1. Reads classification and miRNA mappings. **(A)** Composition of raw reads for each sample. **(B)** Amount of distinct mature miRNAs identified in each sample.

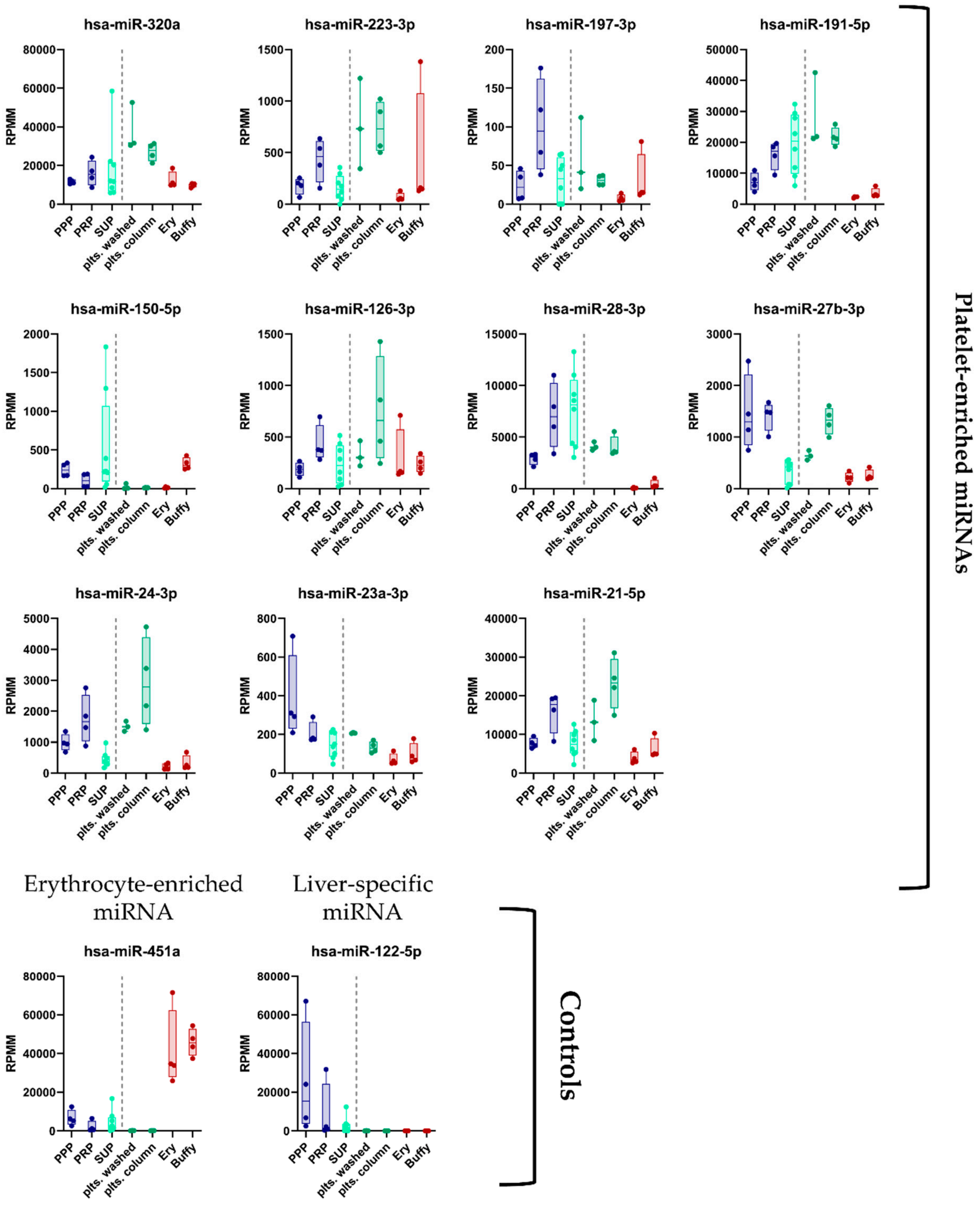

Figure S2. miRNA levels in blood cells and plasma from four healthy volunteers determined by NGS (RPMMs are shown).

Table S1. Comparison of qPCR and NGS data

(* novel biomarker candidates (figure 3))

miRNA	Abundance in Platelets (rank according to qPCR)	Abundance in Platelets (rank according to NGS)
hsa-miR-223-3p	1	10
hsa-miR-21-5p	2	3
hsa-miR-23a-3p	3	12
hsa-miR-191-5p	4	2
hsa-miR-126-3p	5	11
hsa-miR-24-3p	6	7
hsa-miR-451a	7	13
hsa-miR-27b-3p	8	9
hsa-miR-320a	9	1
hsa-miR-151a-5p*	10	6
hsa-miR-199a-3p*	11	4
hsa-miR-197-3p	12	14
hsa-miR-148b-3p*	13	8
hsa-miR-28-3p	14	5
hsa-miR-150-5p	15	16
hsa-miR-122-5p	16	15

A

72 miRNAs identified		
hsa-let-7d-5p	hsa-miR-181c-3p	hsa-miR-485-3p
hsa-let-7e-5p	hsa-miR-181d-5p	hsa-miR-487b-3p
hsa-let-7f-5p	hsa-miR-191-5p	hsa-miR-493-5p
hsa-miR-21-5p	hsa-miR-196b-5p	hsa-miR-548j-5p
hsa-miR-26a-5p	hsa-miR-199a-3p	hsa-miR-584-5p
hsa-miR-26b-5p	hsa-miR-199a-5p	hsa-miR-625-3p
hsa-miR-28-3p	hsa-miR-199b-3p	hsa-miR-654-3p
hsa-miR-28-5p	hsa-miR-221-3p	hsa-miR-654-5p
hsa-miR-30b-3p	hsa-miR-222-3p	hsa-miR-671-3p
hsa-miR-30e-3p	hsa-miR-223-3p	hsa-miR-744-5p
hsa-miR-98-5p	hsa-miR-323b-3p	hsa-miR-766-5p
hsa-miR-126-3p	hsa-miR-328-3p	hsa-miR-889-3p
hsa-miR-127-3p	hsa-miR-340-5p	hsa-miR-1273h-3p
hsa-miR-134-5p	hsa-miR-369-3p	hsa-miR-1277-5p
hsa-miR-139-3p	hsa-miR-370-3p	hsa-miR-1304-3p
hsa-miR-146a-5p	hsa-miR-379-5p	hsa-miR-1307-3p
hsa-miR-146b-5p	hsa-miR-381-3p	hsa-miR-1843
hsa-miR-148b-3p	hsa-miR-382-5p	hsa-miR-1908-5p
hsa-miR-151a-3p	hsa-miR-409-3p	hsa-miR-4433b-3p
hsa-miR-151a-5p	hsa-miR-411-5p	hsa-miR-4433b-5p
hsa-miR-155-5p	hsa-miR-423-3p	hsa-miR-4446-3p
hsa-miR-181a-2-3p	hsa-miR-431-5p	hsa-miR-6852-5p
hsa-miR-181a-3p	hsa-miR-432-5p	hsa-miR-10399-3p
hsa-miR-181c-5p	hsa-miR-433-3p	hsa-miR-11400

B

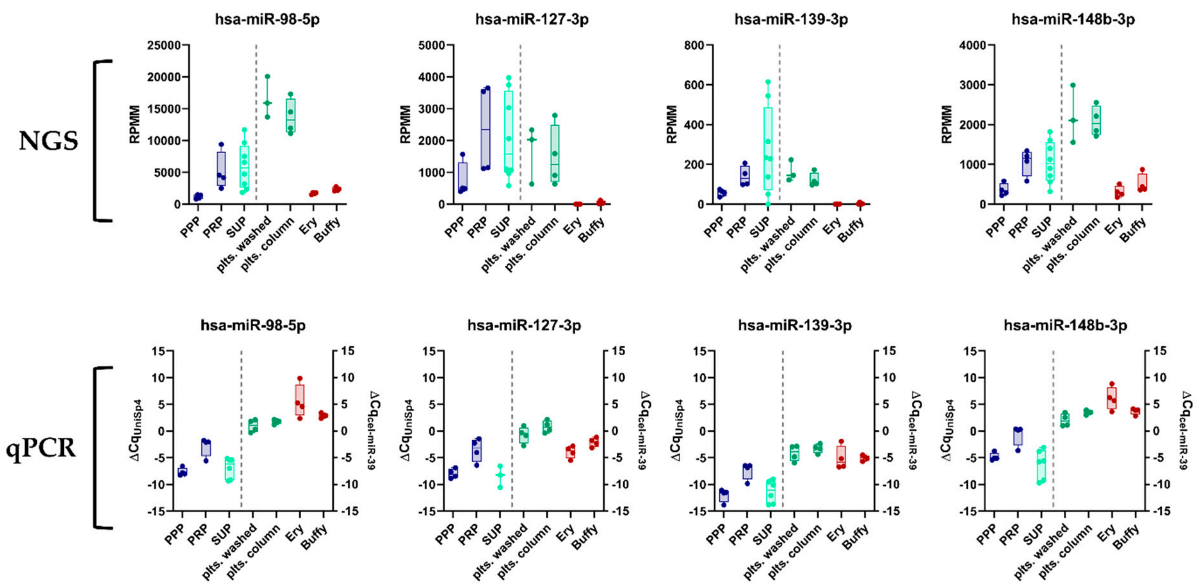

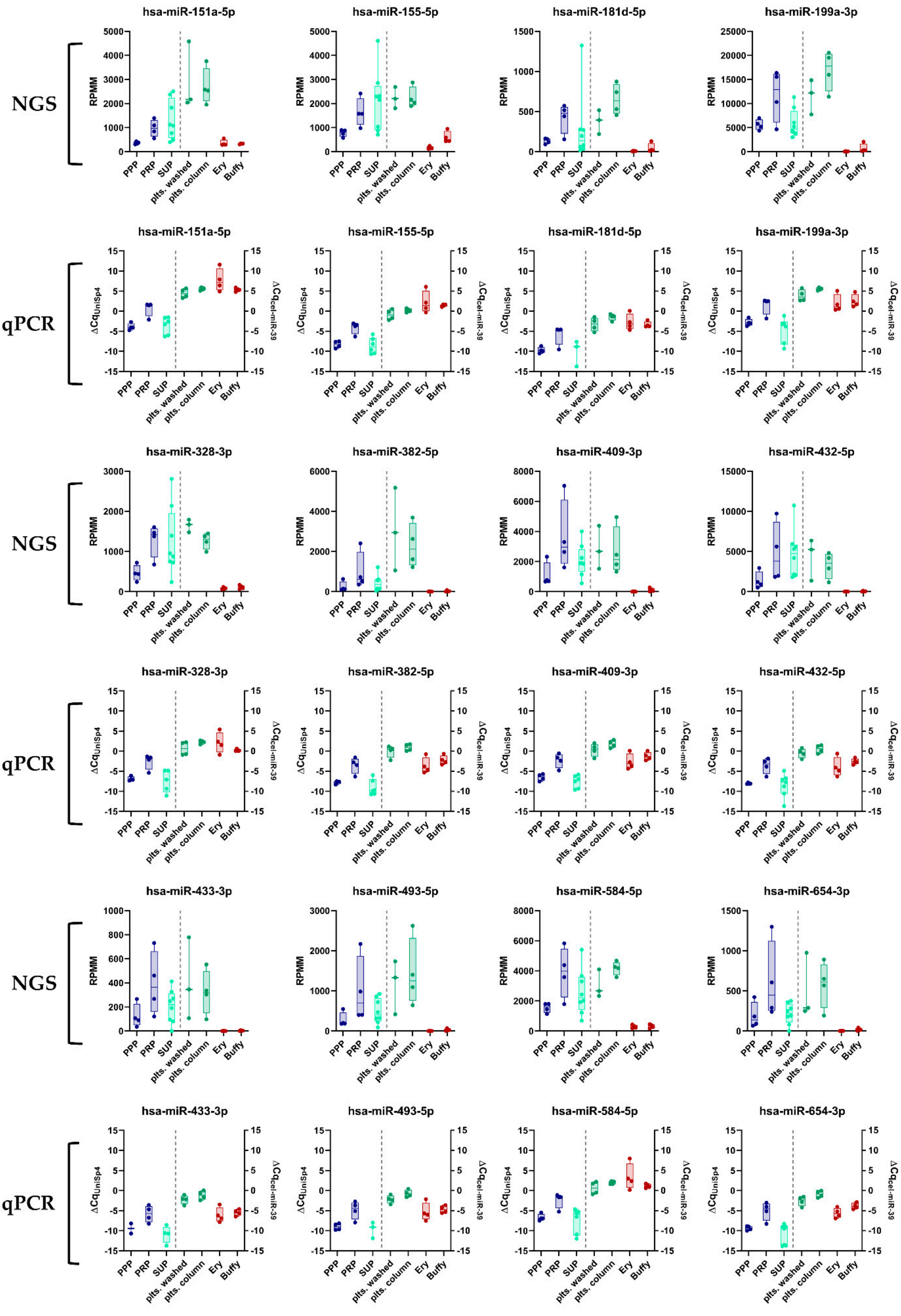

NGS
hsa-miR-151a-5p
hsa-miR-155-5p
hsa-miR-181d-5p
hsa-miR-199a-3p
RPMM
PPP
PRP
SUP
plts. washed
plts. column
Ery
Buffy
qPCR
ΔCq UniSp4
ΔCq cel-miR-39
hsa-miR-328-3p
hsa-miR-382-5p
hsa-miR-409-3p
hsa-miR-432-5p
hsa-miR-433-3p
hsa-miR-493-5p
hsa-miR-584-5p
hsa-miR-654-3p

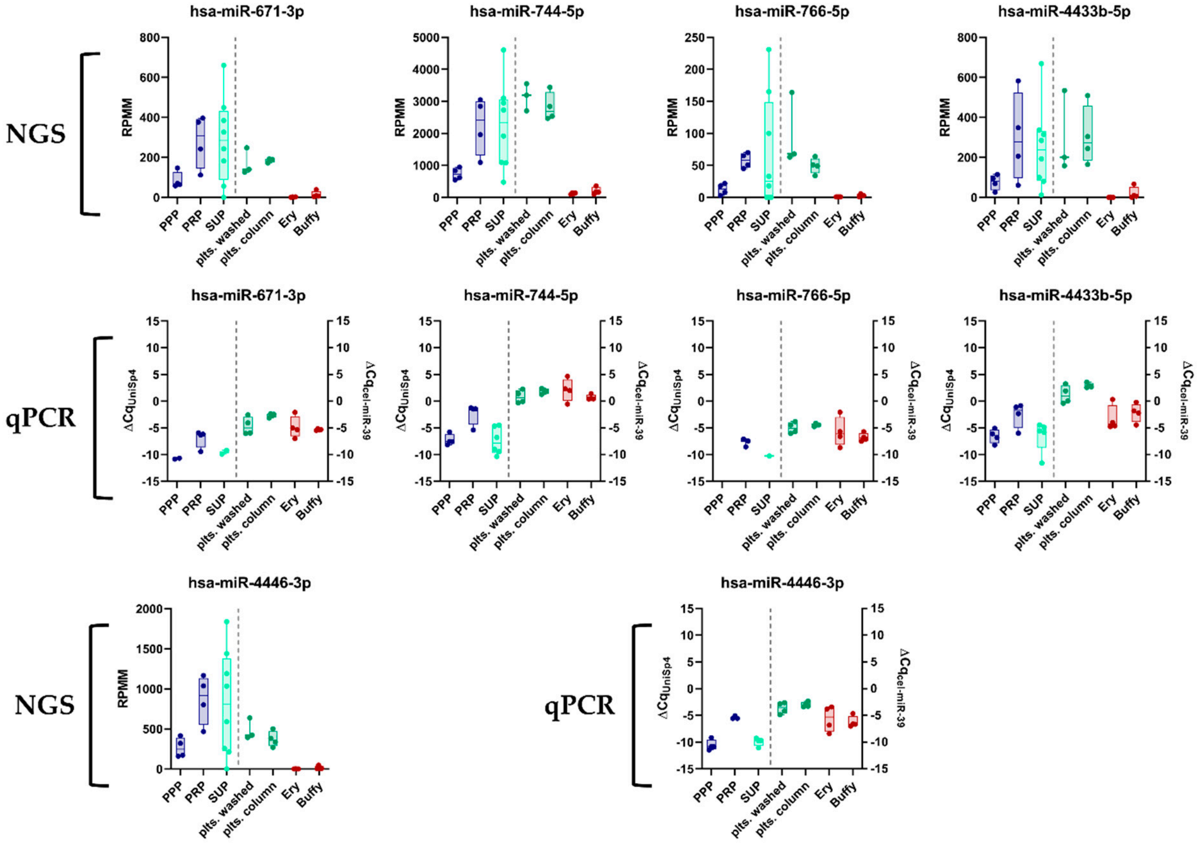

Figure S3. Validation of novel thrombomiR candidates. **(A)** Panel of 72 miRNAs identified by the miRNA processing workflow (blue = miRNAs already part of the established panel, yellow = most promising novel biomarker candidates, grey/yellow = novel biomarker candidates selected for qPCR validation. **(B)** NGS data (RPMM normalized) and qPCR validation of the identified new biomarker candidates. Biofluids are depicted on the left (RNA spike-in normalized), blood cells (cDNA spike-in normalized) on the right side of the plots.

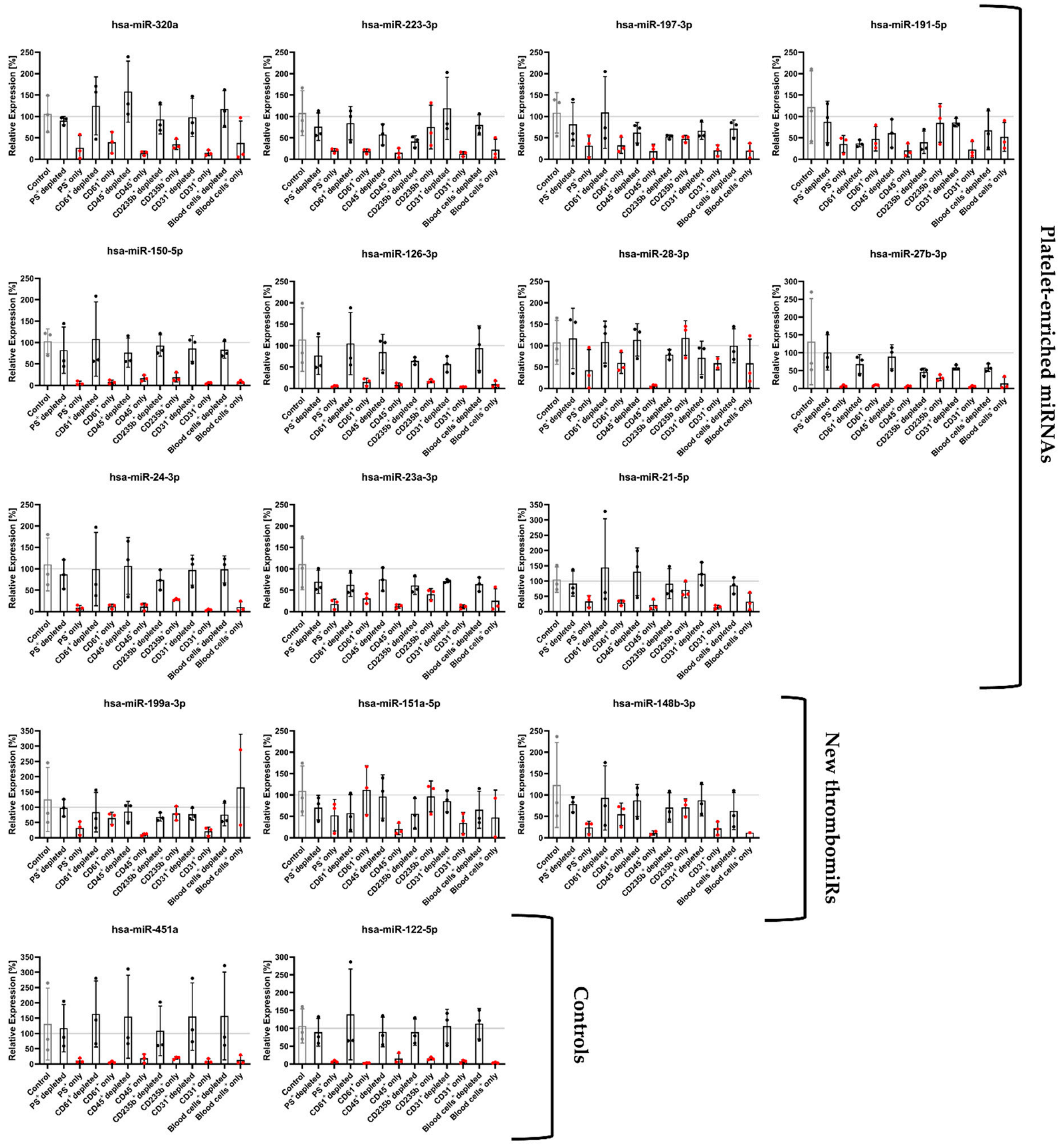

Figure S4. Basal levels of circulatory thrombomiRs in all measured vesicle fractions from three independent donors. Vesicles from total PPP served as control. Relative expression of RNA spike-in normalized values (% of control; linearized ΔΔCqs) is depicted.

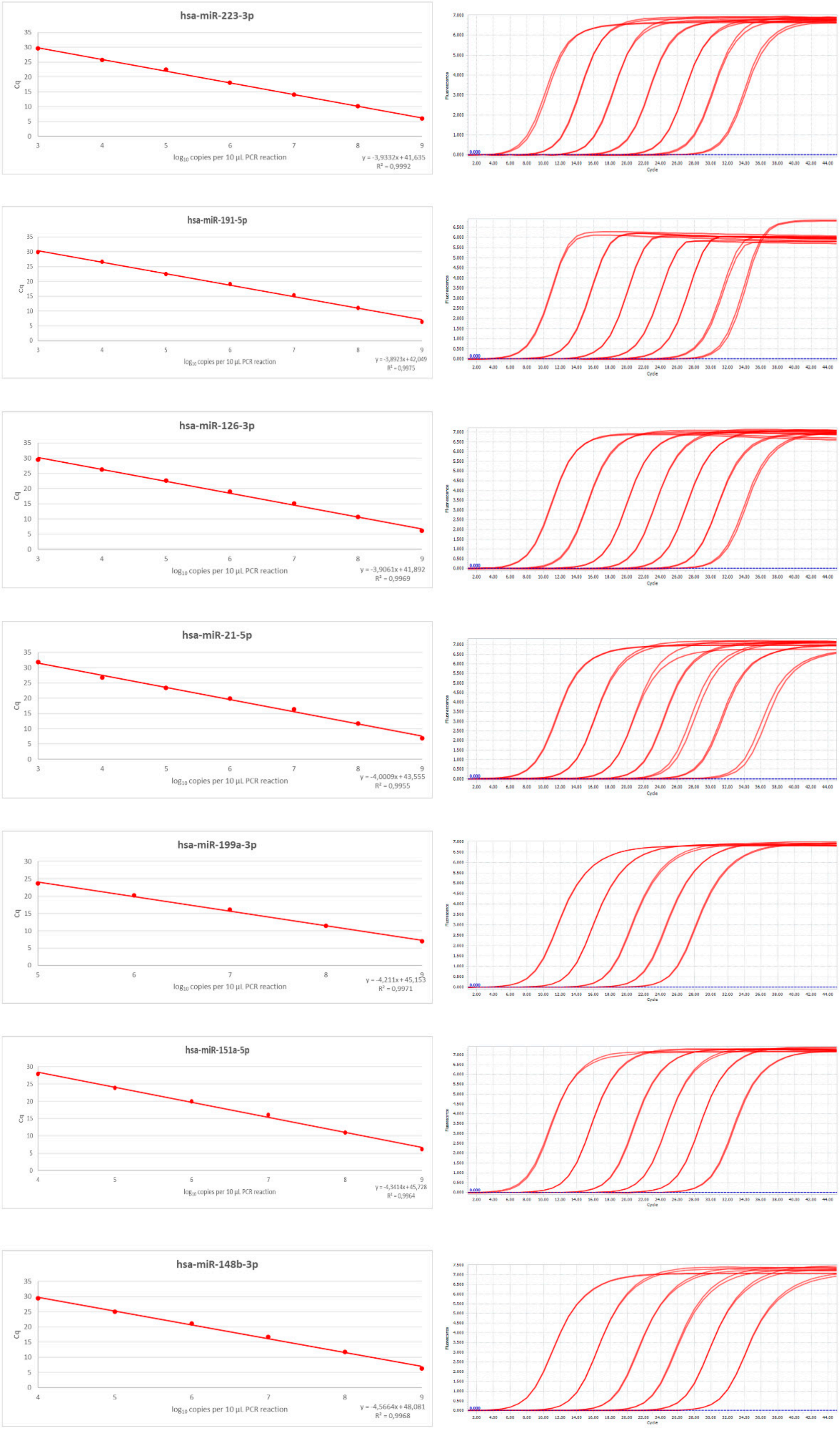

Figure S5. Standard curves and lines fitted to the data of the dilution series of each miRNA. Formulas used for subsequent calculations are displayed in the right corner of each graph.

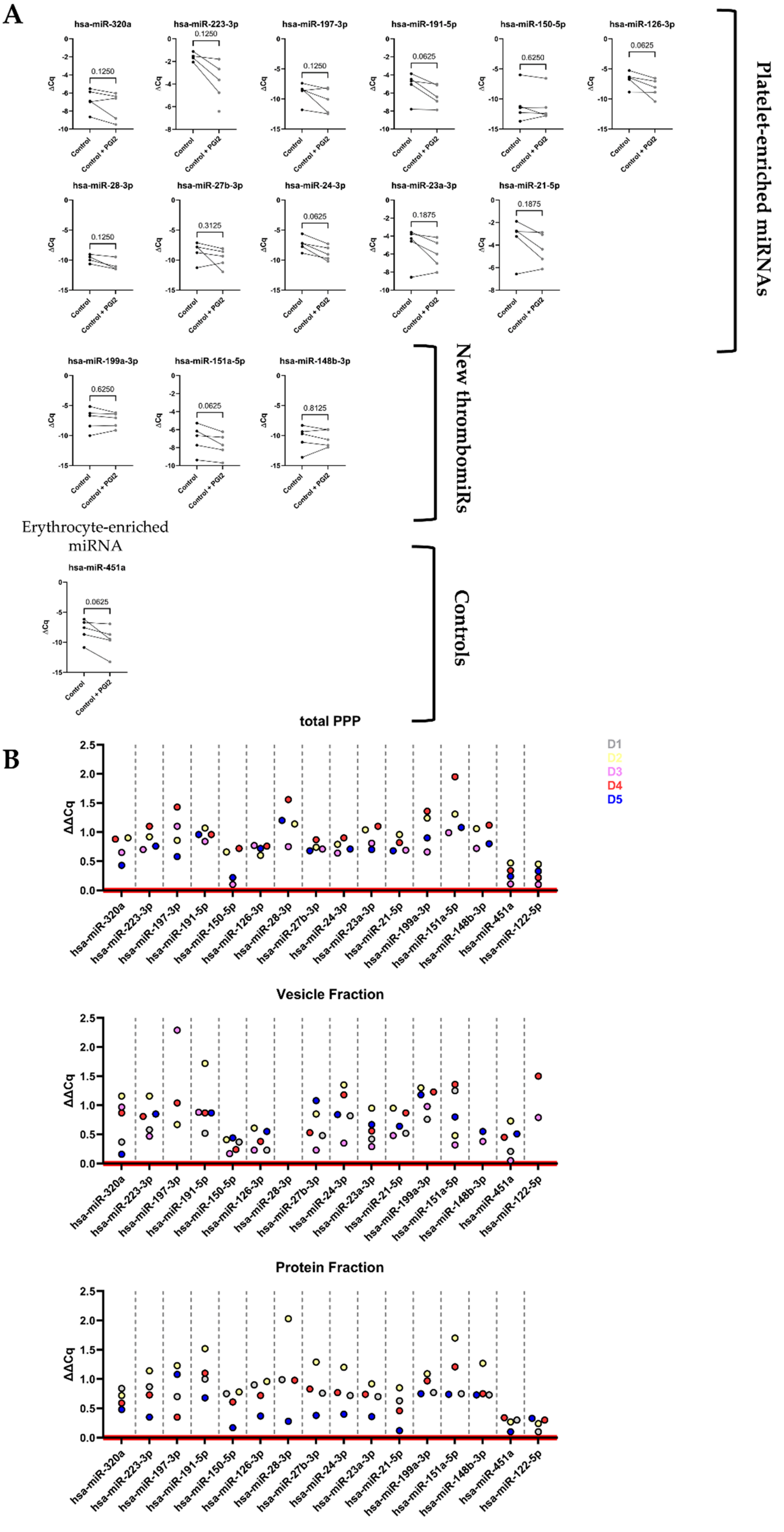
A
hsa-miR-320a
hsa-miR-223-3p
hsa-miR-197-3p
hsa-miR-191-5p
hsa-miR-150-5p
hsa-miR-126-3p
hsa-miR-28-3p
hsa-miR-27b-3p
hsa-miR-24-3p
hsa-miR-23a-3p
hsa-miR-21-5p
Platelet-enriched miRNAs
hsa-miR-199a-3p
hsa-miR-151a-5p
hsa-miR-148b-3p
New thrombomiRs
Erythrocyte-enriched miRNA
hsa-miR-451a
Controls
ΔCq
Control
Control + PGI2
B
total PPP
Vesicle Fraction
Protein Fraction
ΔΔCq
D1
D2
D3
D4
D5
hsa-miR-122-5p

Figure S6. In vitro detection of miRNA secretion upon platelet activation remains challenging. **(A)** Comparison of miRNA levels in the supernatant of unstimulated isolated platelets from five healthy volunteers with or without the additional use of PGI_2 (1 µg/mL) and a reduction of the centrifugation speed during wash steps from 1000 g to 500 g. Wilcoxon matched-pairs signed rank test was calculated. P values <0.05 are considered significant. P values between 0.05-0.2 are depicted. **(B)** Technical variability was assessed by drawing blood from five healthy donors and immediately dividing each sample into two aliquots. All subsequent steps were carried out individually (stimulation of platelets in PRP with 1A5 or ADP; unstimulated control (equal volume of 0.9% NaCl)). SEC was performed using total PPP to isolate the vesicle and protein fractions. Each dot represents the average of Cq differences (ΔΔCq) between technical replicates for each donor. The closer the dots are to y=0, the less variation was introduced through sample handling.

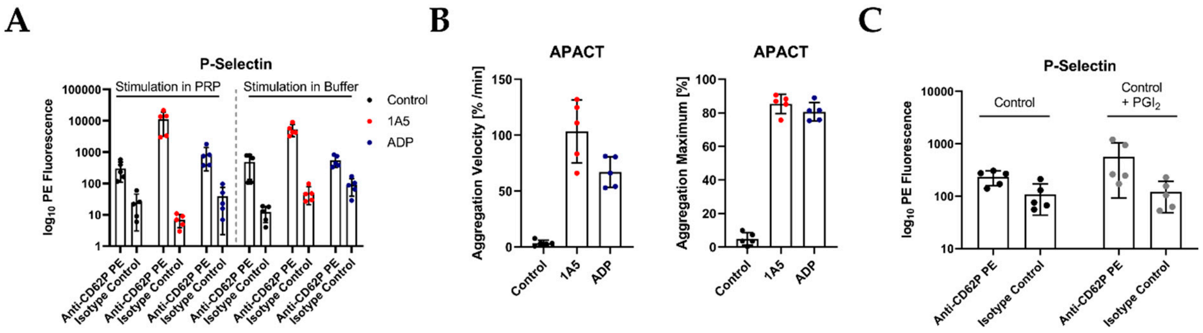

Figure S7. Platelet activation and aggregation markers in the samples of the in vitro experiments (n=5 independent donors). **(A)** P-selectin (CD62P) expression was determined in the platelets used for in vitro stimulation in PRP and buffer. **(B)** Platelet aggregation was measured on an APACT aggregometer with platelets of all donors. **(C)** P-selectin (CD62P) expression was determined on the platelets used to investigate the impact of PGI_2 addition to unstimulated control samples during incubation (S6A) (n=5 independent donors).

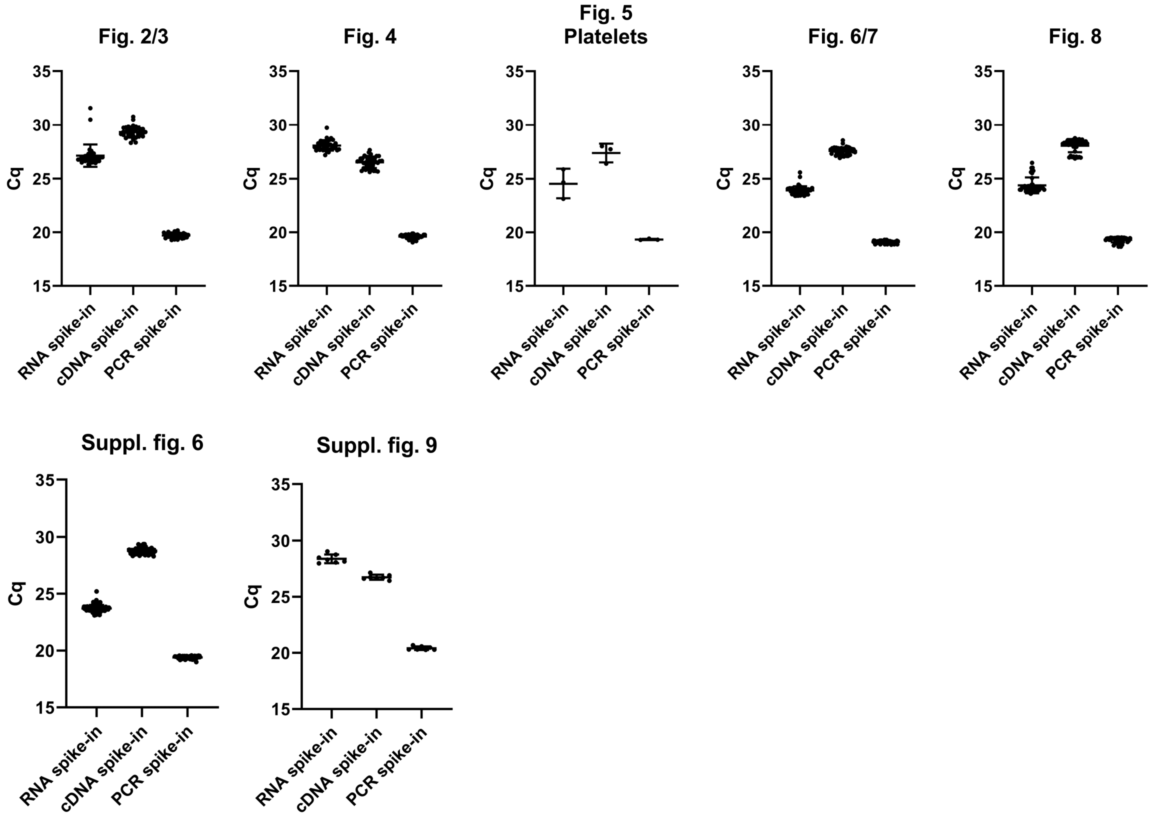

Figure S8. Quality control of all samples. Synthetic spike-ins were added in equimolar amounts before RNA isolation, reverse transcription, and qPCR.

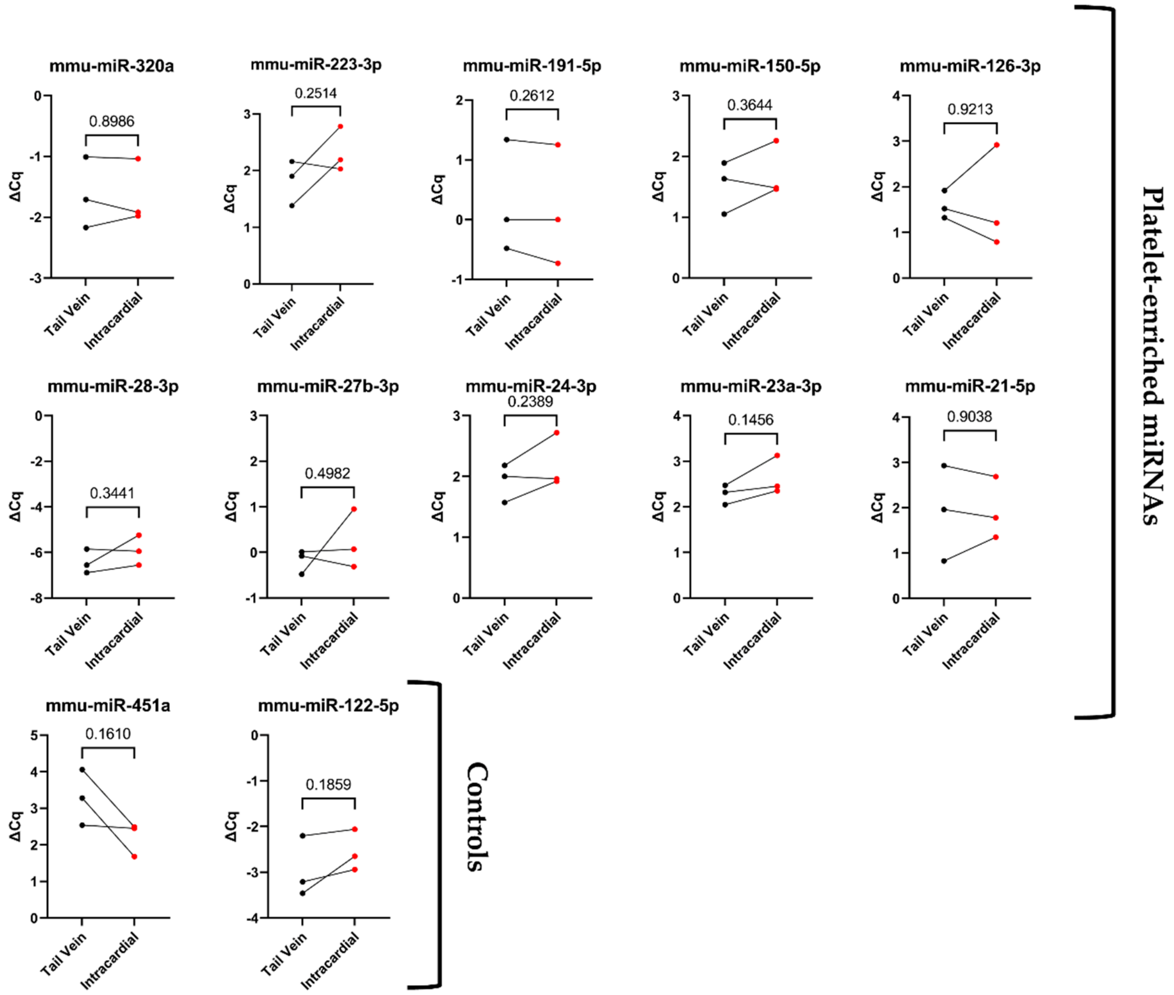

Figure S9. The method of blood withdrawal from mice (n=3) (tail vein vs heart) does not significantly affect thrombomiR levels in PPP. We performed a paired t test. P values <0.05 are considered significant.
